# Projecting terrestrial biodiversity intactness with GLOBIO 4

**DOI:** 10.1111/gcb.14848

**Published:** 2019-11-03

**Authors:** Aafke M. Schipper, Jelle P. Hilbers, Johan R. Meijer, Laura H. Antão, Ana Benítez‐López, Melinda M. J. de Jonge, Luuk H. Leemans, Eddy Scheper, Rob Alkemade, Jonathan C. Doelman, Sido Mylius, Elke Stehfest, Detlef P. van Vuuren, Willem‐Jan van Zeist, Mark A. J. Huijbregts

**Affiliations:** ^1^ PBL Netherlands Environmental Assessment Agency The Hague The Netherlands; ^2^ Department of Environmental Science Institute for Water and Wetland Research Radboud University Nijmegen The Netherlands; ^3^ Centre for Biological Diversity University of St Andrews St Andrews UK; ^4^ Research Centre for Ecological Change Organismal and Evolutionary Biology Research Programme University of Helsinki Helsinki Finland; ^5^ Integrative Ecology Group Estación Biológica de Doñana, Consejo Superior de Investigaciones Científicas (EBD‐CSIC) Sevilla Spain; ^6^ ARIS Utrecht The Netherlands; ^7^ Environmental Systems Analyses Group Wageningen University Wageningen The Netherlands; ^8^ Faculty of Geosciences Utrecht University Utrecht The Netherlands

**Keywords:** anthropocene, biodiversity scenarios, global environmental change, land‐use downscaling, mean species abundance

## Abstract

Scenario‐based biodiversity modelling is a powerful approach to evaluate how possible future socio‐economic developments may affect biodiversity. Here, we evaluated the changes in terrestrial biodiversity intactness, expressed by the mean species abundance (MSA) metric, resulting from three of the shared socio‐economic pathways (SSPs) combined with different levels of climate change (according to representative concentration pathways [RCPs]): a future oriented towards sustainability (SSP1xRCP2.6), a future determined by a politically divided world (SSP3xRCP6.0) and a future with continued global dependency on fossil fuels (SSP5xRCP8.5). To this end, we first updated the GLOBIO model, which now runs at a spatial resolution of 10 arc‐seconds (~300 m), contains new modules for downscaling land use and for quantifying impacts of hunting in the tropics, and updated modules to quantify impacts of climate change, land use, habitat fragmentation and nitrogen pollution. We then used the updated model to project terrestrial biodiversity intactness from 2015 to 2050 as a function of land use and climate changes corresponding with the selected scenarios. We estimated a global area‐weighted mean MSA of 0.56 for 2015. Biodiversity intactness declined in all three scenarios, yet the decline was smaller in the sustainability scenario (−0.02) than the regional rivalry and fossil‐fuelled development scenarios (−0.06 and −0.05 respectively). We further found considerable variation in projected biodiversity change among different world regions, with large future losses particularly for sub‐Saharan Africa. In some scenario‐region combinations, we projected future biodiversity recovery due to reduced demands for agricultural land, yet this recovery was counteracted by increased impacts of other pressures (notably climate change and road disturbance). Effective measures to halt or reverse the decline of terrestrial biodiversity should not only reduce land demand (e.g. by increasing agricultural productivity and dietary changes) but also focus on reducing or mitigating the impacts of other pressures.

## Graphical abstract

We project the changes in terrestrial biodiversity intactness from 2015 to 2050 corresponding with three of the shared socio‐economic pathways combined with different levels of climate change, using an updated version of the GLOBIO model. Biodiversity intactness declined in all three scenarios, with large losses particularly for sub‐Saharan Africa. Effective measures to halt or reverse the decline of terrestrial biodiversity should not only reduce land demand but also reduce or mitigate the impacts of other pressures.

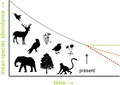

## 1 INTRODUCTION

Global biodiversity is threatened by unprecedented and increasing anthropogenic pressures, including habitat loss and fragmentation, overexploitation, climate change and pollution (IPBES, 2019; Maxwell, Fuller, Brooks, & Watson, 2016; Tilman et al., 2017). This has prompted a proliferation of international commitments and agreements striving to halt biodiversity loss. Prominent examples include the Aichi biodiversity targets (CBD, 2010) and the more recent sustainable development goals (UN General Assembly, 2015), which encompass targets for biodiversity as well as human well‐being, thus underlining that these are interlinked. To deliver on these ambitious goals, decision‐making needs to be supported by a solid understanding of current trends in biodiversity as well as the effects of future changes in drivers and pressures. Scenario‐based biodiversity modelling is indispensable to systematically evaluate the impacts of current and future drivers and pressures on biodiversity and assess the effectiveness of possible conservation measures (IPBES, 2016; Kok et al., 2017; Pereira et al., 2010).

The recently developed shared socio‐economic pathways (SSPs) comprise a set of five diverging plausible future scenarios of human development and associated changes in the environment (O'Neill et al., 2017; Riahi et al., 2017). The SSPs are a combination of qualitative descriptions ('narratives') and model‐based quantifications of potential trends, such as expected human population growth or economic development. The narratives provide the logic and internal consistency of the scenarios and include possible trends in relevant drivers that are more difficult to project quantitatively, such as political stability, environmental awareness and lifestyle (O'Neill et al., 2017; Riahi et al., 2017). The SSPs have already been elaborated in terms of, among others, energy, greenhouse gas emissions and land use (Popp et al., 2017; Riahi et al., 2017). Recently, a protocol has been developed to quantify the SSPs also in terms of biodiversity and ecosystem services, based on harmonized land use and climate change input data and a suite of complementary biodiversity and ecosystem models (Kim et al., 2018), for supporting the global assessment of the Intergovernmental Science‐Policy Platform on Biodiversity and Ecosystem Services (IPBES, 2019). Following this protocol, we assessed the implications of three SSPs for terrestrial biodiversity intactness in 2050. To that end, we used an updated version of the GLOBIO model: a global model of biodiversity intactness, expressed by the mean species abundance (MSA) metric, as a function of multiple anthropogenic pressures on the environment (Alkemade et al., 2009).

An important strength of the GLOBIO model is the breadth of pressures it considers. Originally developed to quantify the impacts of infrastructure on biodiversity intactness (Nellemann et al., 2001), it was later extended to also include the impacts of climate change, land use (via both habitat loss and fragmentation) and atmospheric nitrogen deposition (Alkemade et al., 2009). GLOBIO quantifies biodiversity using the MSA metric, which is a measure of local biodiversity intactness conceptually similar to the biodiversity intactness index (Scholes & Biggs, 2005). For this scenario analysis, we have introduced several new or updated model features to the GLOBIO version as described by Alkemade et al. (2009) and Schipper, Bakkenes, Meijer, Alkemade, and Huijbregts (2016). Because current global‐scale land‐use models are relatively coarse‐grained and tend to underestimate the spatial heterogeneity of land‐use patterns (Hoskins et al., 2016), we have developed a routine to downscale land‐use data to discrete global maps with a spatial resolution of 10 arc‐seconds. This enhances the possibility to account for spatial heterogeneity and ecological effects that depend on the spatial configuration of the landscape—notably habitat fragmentation. Furthermore, the model now allows for quantifying the impacts of hunting in tropical regions, where it is a major pressure (Benítez‐López et al., 2017). Finally, we used updated versions of the modules to quantify the impacts of climate change, land use, habitat fragmentation and atmospheric nitrogen deposition based on updated and extended datasets.

For the scenario analysis, we followed the recently developed biodiversity model intercomparison protocol as described by Kim et al. (2018) by coupling three SSPs (i.e. SSP1, SSP3 and SSP5) with three representative concentration pathways (RCPs; i.e. RCP2.6, RCP6.0 and RCP8.5 respectively), which describe different climate futures based on greenhouse gas emissions throughout the 21st century (van Vuuren et al., 2011). The combinations of SSPs and RCPs allowed us to explore a future with relatively low land‐use change and climate change (SSP1xRCP2.6) as well as futures with high levels of land use or climate change (SSP3xRCP6.0, SSP5xRCP8.5; see Section 2 for further details on the scenarios). We used projections of climate change, land‐use change and atmospheric nitrogen deposition corresponding to each of the three SSPxRCP combinations as inputs to GLOBIO in order to project the changes in terrestrial biodiversity intactness from 2015 to 2050 and identify the main pressures and drivers underlying these changes.

## 2 METHODS

### 2.1 Model description

#### 2.1.1 General approach

**Figure 1:**
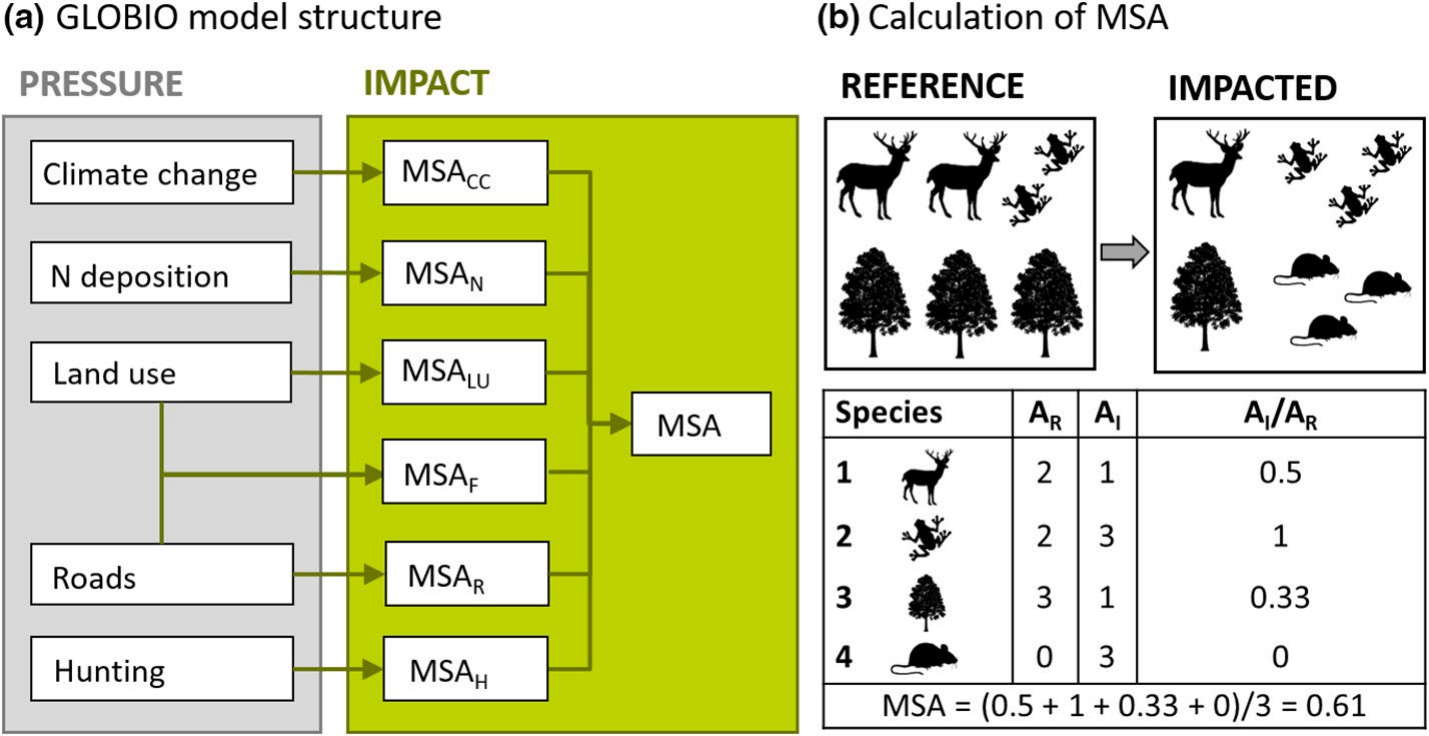
Graphical summary of the GLOBIO model, showing (a) the structure of the model, based on a set of pressure–impact relationships, with CC, climate change; LU, land use; F, fragmentation; R, road disturbance; N, nitrogen deposition; H, hunting; and (b) the calculation of the MSA metric, where IA_R_ denotes individual species' abundance in an undisturbed reference situation, IA_I_ the abundance of the species in the impacted situation, and IA_I_/IA_R_ the truncated abundance ratio, which is calculated only for original species (i.e. occurring in the reference situation)

The core of the GLOBIO model is a set of quantitative relationships that assess the impacts of anthropogenic pressures on biodiversity. Pressures included in GLOBIO are climate change, land use, roads, atmospheric nitrogen deposition and hunting (Figure 1a). Impacts are quantified based on the MSA metric, which is an indicator of local biodiversity intactness (Figure 1b). The metric is quantified based on data that describe changes in community composition in relation to particular pressures. MSA values are retrieved by dividing the abundance of each species found in relation to a given pressure level by its abundance found in an undisturbed situation within the same study, truncating the values at 1, and then calculating the arithmetic mean over all species present in the reference situation (Alkemade et al., 2009; Schipper, Bakkenes, et al., 2016). Increases in individual species abundance from reference to impacted situation are truncated to avoid the indicator being inflated by opportunistic or generalist species that benefit from habitat disturbance. The GLOBIO model combines the pressure–impact relationships with maps of the pressures (i.e. climate change, land use, roads, atmospheric nitrogen deposition and hunters' access points) resulting in maps with impact‐specific MSA values (Figure 1a). Fragmentation impacts are quantified based on the size of natural habitat patches, calculated based on the land use and roads maps. Maps of impact‐specific MSA values are then combined in order to calculate an overall MSA. GLOBIO includes two approaches to integrate MSA values across the impacts, whereby the choice depends on the land use (Table S1). If the land use impact is expected to dominate over other impacts, the overall MSA value equals the MSA value of the land‐use class. For example, it is assumed that there are no additional impacts of atmospheric nitrogen deposition in croplands, which are typically fertilized, and that there are no additional impacts or roads within urban areas. Alternatively, it is assumed that (a) pressures act independently, that is, an organism is lost from the community if at least one of the pressures is higher than its tolerance limit; (b) organisms' tolerances to different pressures are uncorrelated and (c) pressure–impact relationships are based on representative, random samples of the community. Under these assumptions, an overall MSA value is calculated by multiplying the MSA values corresponding with the individual pressures 1 to *m* (Alkemade et al., 2009; Traas et al., 2002), as (Equation 1):

(1) $${\text{MSA}}_{s,i}=\prod_{x=1}^{x=m}{\text{MSA}}_{x,s,i},$$

where MSA*_s_*_,_ *_i_* is the overall MSA for species group *s* in grid cell *i* and MSA*_x_*_,_ *_s_*_,_ *_i_* is the MSA corresponding with pressure *x* on species group *s* in grid cell *i*. Furthermore, the contribution of each pressure to the MSA loss in a given grid cell is calculated relative to the sum of the losses across all pressures and then rescaled to the total loss in the grid cell, as (Equation 2):

(2) $$P_{x,s,i}=\frac{1-{\text{MSA}}_{x,s,i}}{\sum_{x=1}^{x=m}\left(1-{\text{MSA}}_{x,s,i}\right)}\cdot \left(1-{\text{MSA}}_{i}\right),$$

where *P_x_*_,_ *_s_*_,_ *_i_* is the contribution of pressure *x* to the loss in MSA for species group *s* in grid cell *i*. The rescaling is applied to ensure that the sum of the pressure‐specific losses in MSA equals the total loss of MSA in the grid cell. Subsequently, the cell‐specific MSA losses (Equation 1) and pressure contributions (Equation 2) can be aggregated across the grid cells 1 to *n* in any larger region of interest, calculated as mean value weighted by the area of the grid cells.

#### 2.1.2 Pressure–impact relationships

**Figure 2:**
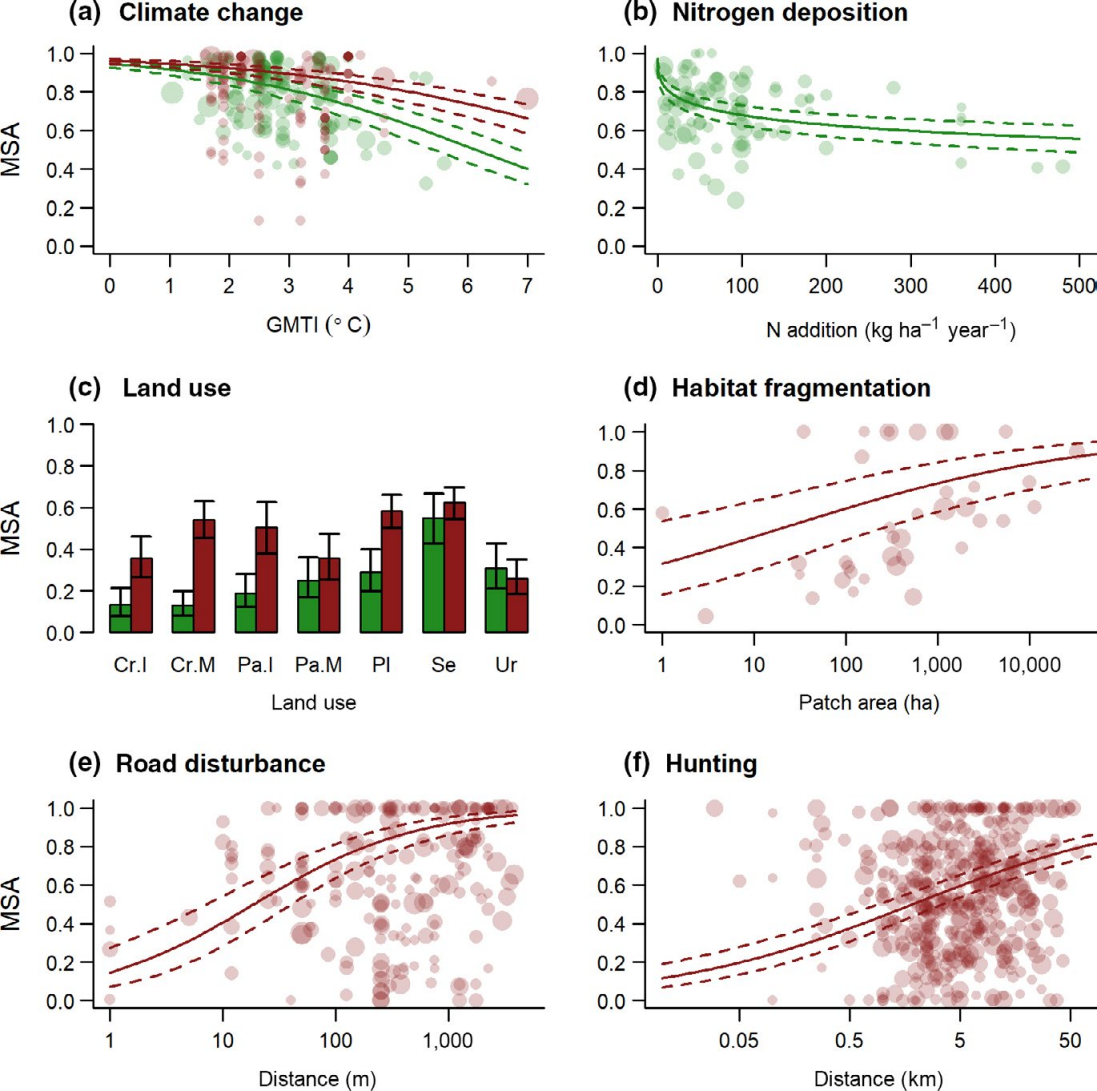
Pressure–impact relationships quantifying mean species abundance (MSA) for plants (green) and warm‐blooded vertebrates (red) in relation to (a) climate change (based on global mean temperature increase), (b) atmospheric nitrogen deposition, (c) land use, (d) habitat fragmentation (based on patch size), (e) disturbance by roads (based on distance to roads) and (f) hunting (based on distance to hunters' access points). Dashed lines and error bars represent the 95% confidence interval. Points represent the individual MSA values with the size reflecting their weight in the model fitting, calculated as the square root of the number of species included in the underlying sample. Land‐use classes include cropland (Cr), pasture (Pa), plantations (Pl), secondary vegetation (Se) and urban (Ur), with M, minimal use and I, intense use [Correction added on 31 December 2019 after first online publication: figure 2C has been updated in this current version.]

To update the pressure–impact relationships in GLOBIO, we used spatially explicit data on species' abundances in relation to different levels or intensities of each pressure (Figure S1). For climate change, nitrogen deposition, road disturbance and hunting, we used databases that were specifically collected for this purpose (Benítez‐López et al., 2017; Benítez‐López, Alkemade, & Verweij, 2010; Benítez‐López, Santini, Schipper, Busana, & Huijbregts, 2019; Midolo et al., 2019; Nunez, Arets, Alkemade, Verwer, & Leemans, 2019). For land use and habitat fragmentation, we used the PREDICTS database, which includes spatial comparisons of species' assemblages among different land‐use types and habitat patch sizes (Hudson et al., 2017). For each pressure, we collected or selected the data such that influences of other pressures could be considered negligible or equal between control and treatment. For each dataset and pressure level or intensity, we first calculated species‐specific abundance ratios by dividing each species' abundance in the disturbed situation by its abundance in the corresponding reference site, and then retrieved MSA values by averaging the truncated abundance ratios. Next, we established mixed effect beta regression models with logit link function to relate the MSA values to the pressure gradient, whereby we used dataset within study as nested random intercept to account for possible non‐independence of observations as well as possible systematic differences among datasets. Because beta regression requires input data to be restricted to the closed interval [0,1], we applied a Smithson–Verkuilen transformation to the MSA values for a given pressure if the set included zeros or ones (Smithson & Verkuilen, 2006). Because MSA is an assemblage‐level metric, we weighted the observations based on the number of species sampled (square‐root transformed to reduce the skewness in the data). In this study we included impact relationships for terrestrial plants and warm‐blooded vertebrates (birds and mammals), because the majority of the monitoring data is on these two species groups. For each group, we included only the impacts assumed to be the most relevant, that is, we included climate change, nitrogen deposition and land use for plants, and climate change, land use, infrastructure disturbance, fragmentation and hunting for warm‐blooded vertebrates (Figure 2). Where possible based on the data available, we tested for the influence of potential moderators on the impact relationships (e.g. the influence of climate zone on the climate change impact relationships) and identified the most parsimonious model based on the Bayesian information criterion (BIC). We preferred BIC over alternative approaches to model selection (e.g. Akaike information criterion) in order to minimize the risk of overfitting. We performed all data processing and model fitting in the R environment (R Core Team, 2017), including the glmmTMB package for beta regression modelling (Brooks et al., 2017). Further methodological details on the fitting of the specific pressure–impact relationships are provided in Text section S1.

#### 2.1.3 Land‐use downscaling

Because current global land‐use models are relatively coarse‐grained and tend to underestimate the spatial heterogeneity of land‐use patterns (Hoskins et al., 2016), we have extended GLOBIO with a routine to downscale coarse‐grain land‐use data to discrete maps with a spatial resolution of 10 arc‐seconds. The land‐use downscaling procedure requires three inputs: regional totals or demands (‘claims’) of each land‐use type; map layers quantifying the suitability of each grid cell for each land‐use type; and a ‘background’ map defining the land cover or land use of cells that are not being converted for fulfilling the claims. Claims can be derived from national or regional statistics or from models that estimate demands for land based on socio‐economic developments, such as integrated assessment models. All claims need to be expressed in terms of area (km^2^). Suitability layers reflect the relationships between the occurrence of the land‐use types and relevant environmental covariates, which may include existing land‐use patterns, physiographic variables and variables indicative of access to infrastructure. The allocation algorithm prioritizes candidate grid cells according to their suitability values and allocates the claims of each land‐use type in each region starting from the cells with the highest suitability until the total claim is allocated. In the allocation procedure, GLOBIO follows a predefined order in which urban land is allocated first, followed by cropland, reflecting that urbanization is typically prioritized at the expense of other land‐use types (including existing cropland; Bren d'Amour et al., 2016; Liu et al., 2019), while cropland expansion often takes place in forest or grazing land (Piquer‐Rodríguez et al., 2018). Forestry and pasture are allocated thereafter, such that forestry is allocated within remaining forest areas, and reflecting that grazing typically takes place in areas not productive enough for crops (Hasegawa, Fujimori, Ito, Takahashi, & Masui, 2017). If for a given land‐use type in a given region multiple cells have equal suitability, the land‐use claim is distributed randomly among those cells. Claims or changes in claims relative to a preceding scenario–year are allocated per scenario–year combination. If the land claim allocated in a given scenario–year is smaller than the claim allocated in the preceding scenario–year, cells are abandoned in reverse order of suitability and assigned to secondary vegetation.

### 2.2 Application: SSP projections

#### 2.2.1 Scenarios

Following the recently developed biodiversity model intercomparison protocol (Kim et al., 2018), we used three SSPs associated with different levels of human pressure on the environment: SSP1 (‘sustainability’), SSP3 (‘regional rivalry’) and SSP5 (‘fossil‐fuelled development’). The sustainability scenario is characterized by a relatively low population growth, low growth in consumption due to less resource‐intensive lifestyles (e.g. less meat) and more resource‐efficient technologies, increased regulation of land‐use change due to expansion of the protected area network, and substantial improvements in agricultural productivity, allowing for reforestation. The regional rivalry scenario is characterized by high population growth, resource‐intensive consumption, low agricultural productivity and limited regulation of land‐use change, leading to continued deforestation. Finally, the fossil‐fuelled development scenario is characterized by low population growth, strong economic growth, a consumption‐oriented and energy‐intensive society, and highly intensive agricultural practices leading to a decline in deforestation. We combined the SSPs with climate projections according to the RCPs such that the combinations covered a broad range of land‐use and climate change, following the biodiversity model intercomparison protocol (Kim et al., 2018). Thus, we linked SSP1 (moderate land‐use pressure) with RCP2.6 (low level of climate change), SSP3 (high land‐use pressure) with RCP6.0 (moderate level of climate change) and SSP5 (moderate land‐use pressure) with RCP8.5 (high level of climate change). The SSP3xRCP6.0 and SSP5xRCP8.5 combinations represent the so‐called baseline scenarios, that is, scenarios including only modest or even no climate change mitigation policy. In contrast, SSP1xRCP2.6 includes mitigation measures, for example reforestation and bioenergy production, to achieve the radiative forcing level of RCP2.6, consistent with 2 degree warming (van Vuuren et al., 2017). We projected the biodiversity implications of each SSPxRCP combination from 2015 to 2050, using input data on the relevant pressures as further described below, and aggregated the results to 17 world regions as distinguished by the IPBES (Brooks et al., 2016).

#### 2.2.2 Pressure input data

We retrieved the global mean temperature increase since 1970 (in °C) for 2015 and for each selected RCP for 2050 from the MAGICC climate model, which is part of the IMAGE model framework (Meinshausen, Raper, & Wigley, 2011; Stehfest et al., 2014). We retrieved nitrogen deposition data (kg ha^−1^ year^−1^; 0.5° resolution) for each scenario–year combination also from IMAGE. To compile the land‐use maps, we used the newly implemented land‐use allocation module. We first established suitability layers for urban land and cropland based on the distance to existing urban and cropland areas, for pasture based on livestock densities, and for forestry based on existing forest cover, elevation and distance to roads and rivers (Text section S2). The suitability of natural land cover within protected areas was set to zero in order to limit land‐use expansion within these areas. Next, we compiled a land‐use map for 2015 using as background map the ESA climate change initiative land‐cover map for 2015 (ESA, 2017), which already includes urban area and cropland, and by downscaling country‐level areas of pasture and forestry land as reported by the FAO (Text section S2). We note that the resulting present‐day land‐use map does not include secondary vegetation as the background land‐cover map does not distinguish between primary and secondary vegetation. For compiling the land‐use maps for 2050, we used the same suitability layers and background map as for the present‐day land‐use map and downscaled SSP‐specific country‐level claims of urban land, cropland, pasture (pasture and rangeland) and forestry (based on wood harvest), which we retrieved from the land‐use harmonization dataset version 2 (LUH2; http://luh.umd.edu/data.shtml ). After the allocation, we differentiated cropland and pasture into different use intensity classes based on nitrogen application rates, also retrieved from LUH2 (Text section S2). We retrieved data for roads from the recently released dataset resulting from the global road inventory project (GRIP; Meijer, Huijbregts, Schotten, & Schipper, 2018). We assumed that impacts due to roads are caused by highways, primary roads and secondary roads only (road types 1–3 in GRIP), excluding road types 4 and 5 because minor roads induce much less avoidance behaviour in wildlife (Brehme, Tracey, Mcclenaghan, & Fisher, 2013). To account for expected future increases in traffic (Dulac, 2018), we assumed that the smaller road types 4 and 5 would be transformed into main roads (and hence contribute to the impacts) in the future scenarios. In the fragmentation module, we defined fragments as neighbouring cells (8‐neighbour rule, i.e. including cells on the diagonal) of primary or secondary vegetation dissected by a road or by cropland, urban or intensively used pasture area. We excluded minimally used pasture from causing fragmentation because extensive grazing typically takes place within the existing vegetation (Alkemade, Reid, Berg, Leeuw, & Jeuken, 2013). We obtained the distance to settlements, needed as input to quantify hunting impacts in the tropics, as the Euclidean distance to the nearest village within tropical biomes. We delineated tropical biomes based on the biomes map compiled by Dinerstein et al. (2017) and retrieved locations of villages by merging data from OpenStreetMap (http://download.geofabrik.de), the Humanitarian Data Exchange (http://www.data.humdata.org) and national databases. Because hunting takes place from small, rural villages, we filtered out settlements that coincided with cells classified as urban area on the land‐use maps (Benítez‐López et al., 2019). Because of a lack of information on new future settlements, we used the present‐day settlement data also for the scenario projections. An overview of the extent to which the pressure input data are covered by the data used to fit the pressure–impact relationships is provided in Figure S2.

## 3 RESULTS

### 3.1 Projected biodiversity changes

**Figure 3:**
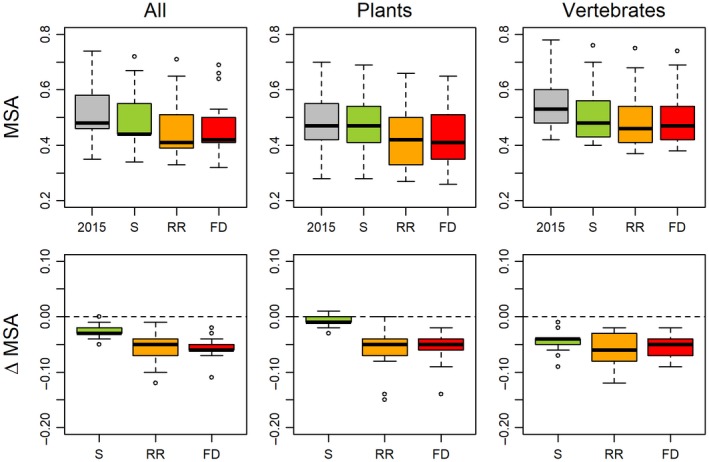
Variation in area‐weighted mean species abundance (MSA; top) and changes in MSA (bottom) from 2015 to 2050 per scenario across 17 IPBES regions for both taxonomic groups (left), plants (centre) and warm‐blooded vertebrates (right). Overall values represent the mean across plants and warm‐blooded vertebrates. S, sustainability scenario (SSP1xRCP2.6); RR, regional rivalry scenario (SSP3xRCP6.0) and FD, fossil‐fuelled development scenario (SSP5xRCP8.5). Underlying region‐ and scenario‐specific MSA values are provided in Table S2; 5th and 95th percentiles per IPBES region are given in Table S3

**Figure 4:**
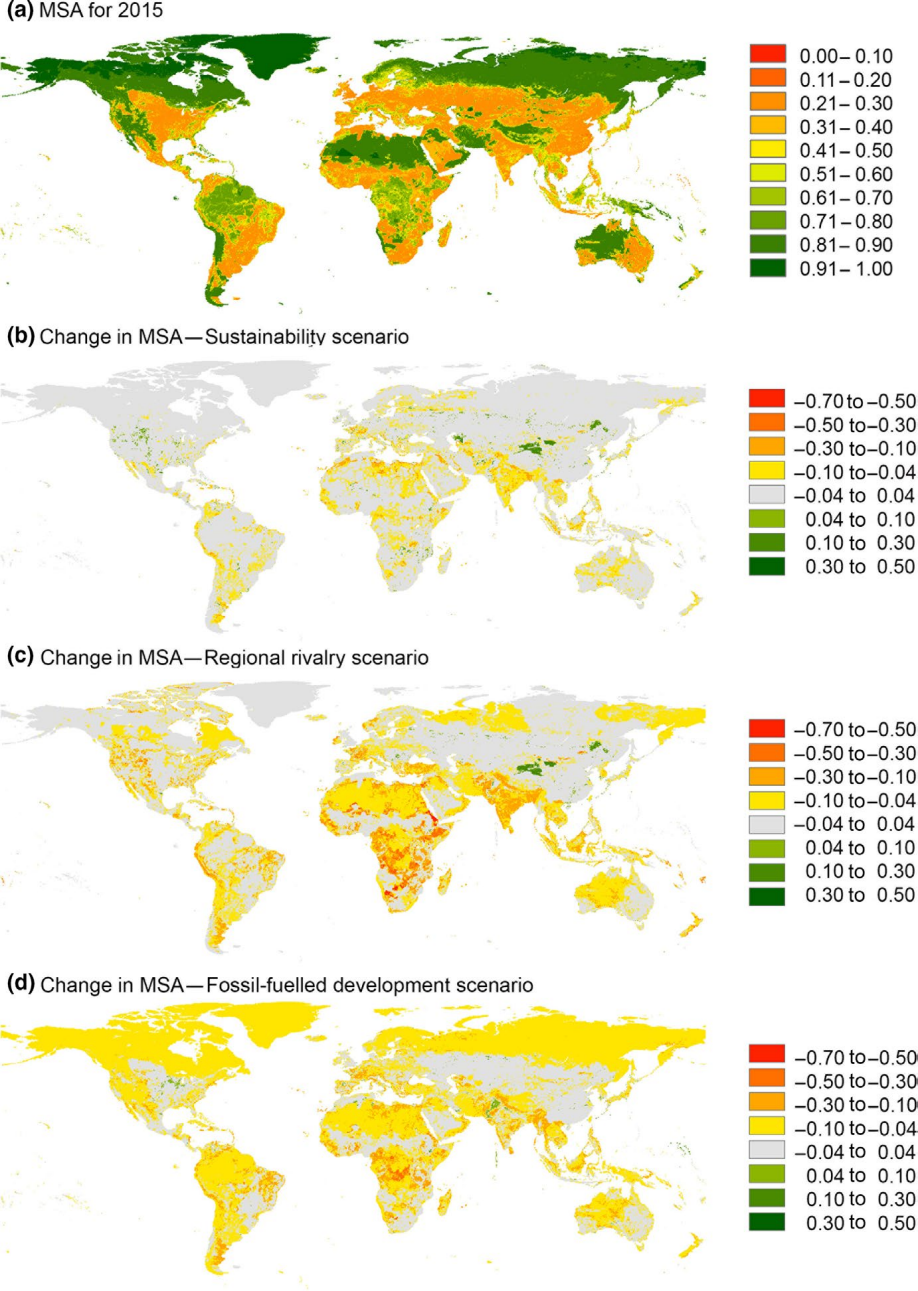
Global patterns in (a) mean species abundance (MSA) values for 2015 and changes in MSA values from 2015 to 2050 for (b) SSP1xRCP2.6, (c) SSP3xRCP6.0 and (d) SSP5xRCP8.5. Values represent the mean across plants (Figure S3) and warm‐blooded vertebrates (Figure S4). For visualization purposes, the maps were resampled to a resolution of 0.25 degree based on the mean across the underlying values

For 2015, we estimated a global area‐weighted mean MSA of 0.56 (Table S2). Future projections resulted in an overall decrease in MSA for all three scenarios (Figure 3; Table S2). The global area‐weighted mean MSA value was projected to decline by 0.02 in the sustainability scenario (SSP1xRCP2.6), by 0.06 in the regional rivalry scenario (SSP3xRCP6.0) and by 0.05 in the fossil‐fuelled development scenario (RCP5xRCP8.5) (Table S2). To put these numbers in perspective, losses of 0.02–0.06 in global mean MSA are equivalent to roughly 2.5–8 million km^2^ of pristine habitat (i.e. MSA = 1) being converted to habitat where all the original species are extirpated (i.e. MSA = 0). This is equivalent to an area ranging from one‐third to the entire size of Australia. The sustainability scenario was more beneficial to plants than to vertebrates (i.e. smaller global area‐weighted mean losses in MSA), whereas the reverse was true for the other scenarios (Figure 3; Table S2). Our projections further revealed clear spatial variation in biodiversity change (Figures 3 and 4; Figures S3 and S4; Tables S2 and S3). On average, we found the largest projected declines for East Africa, Central Africa and Southern Africa in the regional rivalry scenario. The smallest declines occurred in North‐East Asia (sustainability and regional rivalry scenarios) and North‐America (sustainability scenario). Spatial patterns for plants and warm‐blooded vertebrates were largely similar, although in the regional rivalry and fossil‐fuelled development scenarios we found larger declines in MSA for plants than for vertebrates particularly in the boreal and Arctic regions (Figures S3 and S4).

### 3.2 Pressure contributions

**Figure 5:**
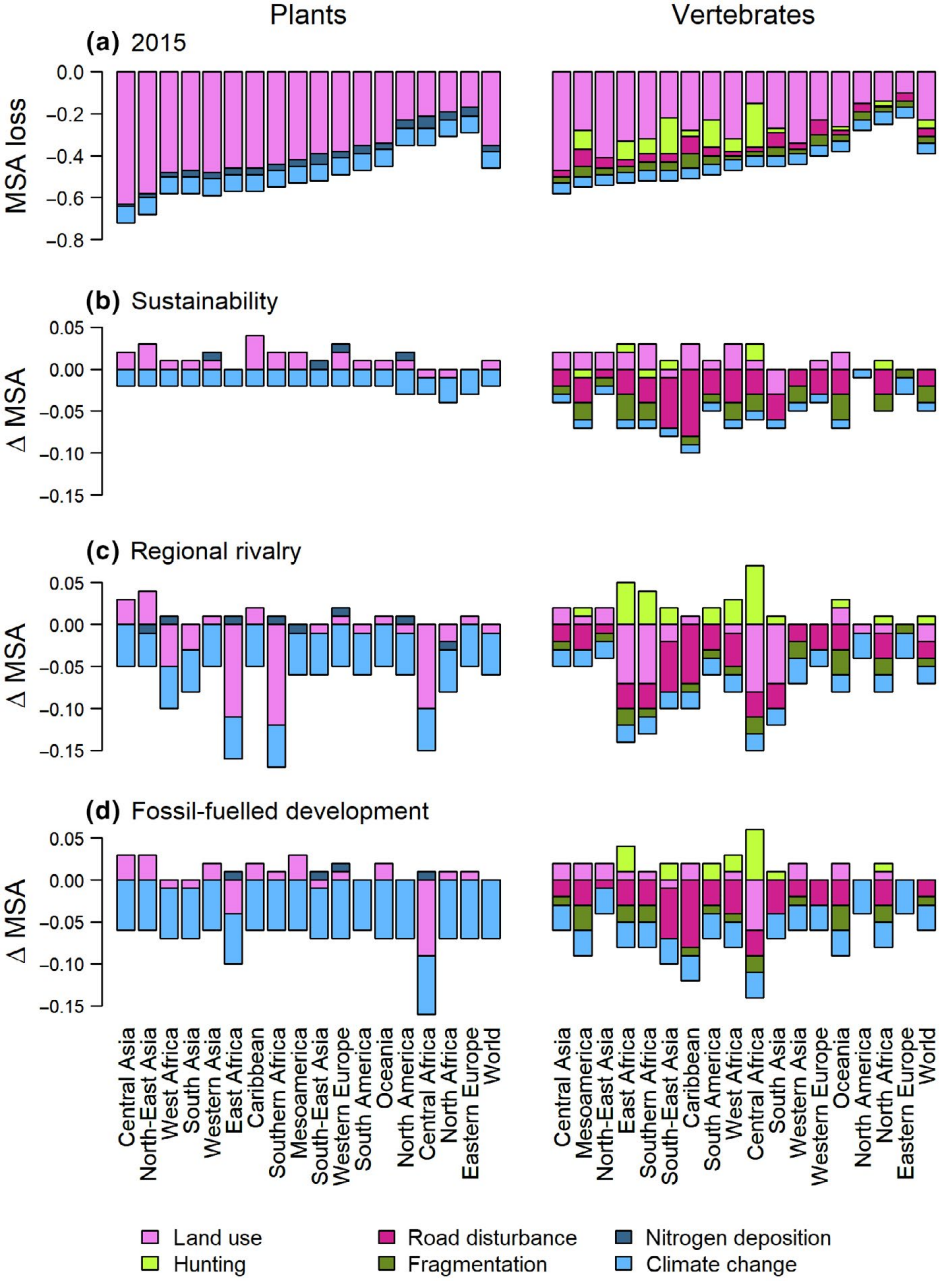
Losses in mean species abundance (MSA) per pressure and per IPBES subregion and globally for plants (left) and warm‐blooded vertebrates (right) for 2015 (a) and changes in MSA resulting from changes in each pressure in each of the three future scenarios in 2050 (b–d). Underlying numbers are provided in Table S4

For plants, the global loss in MSA as calculated for 2015 was mainly related to land‐use change (area‐weighted global mean MSA loss of –0.35), followed by climate change (–0.08) and atmospheric nitrogen deposition (–0.03; Figure 5; Table S4). This ranking of pressures was consistent across regions. For vertebrates, the global loss in MSA in 2015 was also mostly related to land use, with an area‐weighted global mean MSA loss of –0.23. Influences of the other pressures were considerably smaller, with area‐weighted global mean MSA losses ranging from –0.03 for fragmentation to –0.05 for climate change. Land use was the dominant pressure for vertebrates in all regions, expect in Central Africa where its impact was exceeded by hunting. Although land use remained the most important pressure in the future scenarios, for both plants and vertebrates (Figure S5), we observed various changes in pressure contributions. The impacts of climate change consistently increased, with larger increases for higher levels of radiative forcing (Figure 5). Impacts of infrastructure disturbance and fragmentation on vertebrates increased as well, as a result of the projected increase in use intensity of the global road network. Impacts of hunting typically decreased, reflecting reductions in the total area of natural vegetation, where hunting occurs, as well as declines in the number of rural settlements (which may disappear because of urbanization; Table S5). Changes in the impacts of land use and nitrogen deposition showed clear spatial variability, with impacts decreasing in some scenario‐region combinations and increasing in others (Figure 5). Large increases in land‐use impacts were projected for Central Africa, East Africa and Southern Africa in the regional rivalry scenario. In the sustainability scenario, the majority of the regions was characterized by a decline in land‐use impacts.

## 4 DISCUSSION

### 4.1 Global trends

Our projections indicate that biodiversity intactness will decline from present‐day to 2050, even in the most optimistic scenario evaluated. These declines comply with a mid‐term analysis of progress towards the Aichi biodiversity targets for 2020, which revealed that pressure indicators were mostly on a continuing increasing trend, while biodiversity indicators pointed to a continuing decline (Tittensor et al., 2014). Projected area‐weighted global mean losses in MSA were similar for the regional rivalry (SSP3xRCP6.0) and fossil‐fuelled development (SSP5xRCP8.5) scenarios. Compared to the fossil‐fuelled development scenario, the regional rivalry scenario is characterized by a larger increase in global human population and a smaller increase in agricultural production efficiency (KC & Lutz, 2017; Popp et al., 2017), leading to an overall higher demand for agricultural land (Table S5). However, climate change impacts were larger in the fossil‐fuelled development scenario, due to larger projected increases in global mean temperature, leading to similar overall biodiversity losses in the two scenarios, albeit via different pressures (Figure 5). The results of the sustainability scenario (SSP1xRCP2.6) indicate that biodiversity declines may slow down in response to a decreasing demand for agricultural land. The sustainability scenario is characterized by a global decline of ~5% in agricultural land area in 2050 (Table S5), despite an increase in the global human population relative to the present‐day (KC & Lutz, 2017). This decline in agricultural land reflects increases in agricultural productivity combined with altered consumption patterns (30% reduction of animal products consumption) and a reduction of food losses in both the supply chain and households (33% reduction of food waste; Doelman et al., 2018). The reduction of agricultural land results in an increase in secondary vegetation area (Table S5), which is characterized by higher MSA values than the agricultural land‐use types (Figure 2), thus yielding a partial restoration of biodiversity intactness. Interestingly, the global human population projected for 2050 is highly similar between the sustainability scenario (8.5 billion people) and the fossil‐fuelled development scenario (8.6 billion people; KC & Lutz, 2017), whereas the latter is characterized by an increase rather than a decline in agricultural land area (+2.5% worldwide; Table S5). The comparison between these two scenarios thus highlights the importance of changes in both production and consumption of agricultural products in order to limit the environmental impacts, in line with the results of other recent studies (Di Marco et al., 2019; Erb et al., 2016; Springmann et al., 2018). Yet, alongside relatively stable or reduced land demand in the sustainability scenario, we found clear increases in the impacts of climate change and road disturbance (Figure 5). The increased impacts of climate change with increasing levels of radiative forcing was particularly evident for plants, reflecting the steeper slope of the pressure–impact relationship (Figure 2), as well as the smaller number of pressures included for plants as compared to warm‐blooded vertebrates. Our results thus highlight that reducing agricultural land demand alone will not be sufficient to halt or revert the global decline of biodiversity if not accompanied by measures to reduce or mitigate other pressures.

### 4.2 Pressure contributions

We found that land use is currently the dominant pressure on terrestrial biodiversity, exceeding the present‐day impacts of hunting, climate change and pollution. This is line with other recent analyses that ranked pressures affecting community composition and species' populations (IPBES, 2019; Maxwell et al., 2016; Newbold, 2018). We note that our assessment may underestimate the present‐day impacts of hunting because there might be more settlements and other relevant hunters' access points than included in our input data. Moreover, we assessed the hunting impacts only for the tropics, due to a lack of data to include other regions. On the other hand, the pressure–impact relationship for hunting may overestimate the impacts because the underlying observations are biased towards medium‐ and large‐sized species, which comprise the majority of our data (Text section S1), and which are more heavily hunted than small‐sized species (Benítez‐López et al., 2017; Ripple et al., 2016). Impacts of fragmentation might be underestimated because our pressure–impact relationship assumes that such impacts are absent in natural habitat patches larger in size than 10,000 ha (see Text section S1), due to insufficient biodiversity monitoring data including larger reference patches. Although 10,000 ha might be large enough to fulfil the minimum area requirements of small and herbivorous bird and mammal species, it is likely too small for minimum viable populations of large carnivores (Pe'er et al., 2014), and hence a fully intact community. This implies that the effects of fragmentation, and thus land use as one of the underlying causes, could be larger than assessed here.

Our scenario projections suggest that land use will also be the most important cause of biodiversity loss in 2050. This is consistent with the projections of Sala (2000), but in contrast to studies indicating that impacts of climate change on biodiversity may have exceeded land‐use impacts halfway this century (Di Marco et al., 2019; Newbold, 2018). It is notoriously difficult to quantify the effects of future climate change in comparison to the impacts of other threats, reflecting model as well as data limitations (Newbold, 2018; Tingley, Estes, & Wilcove, 2013). The pressure–impact relationships used in this study are based on relative species richness estimates retrieved from bioclimatic envelope modelling results rather than observational data of MSA (Text section S1), due to a lack of local biodiversity monitoring data across sufficiently wide climate gradients. Moreover, we considered global mean temperature increase only, thus ignoring the possible changes in seasonality or extremes as well as latitudinal differences in the magnitude of climatic change. More research on the effects of climate change on biodiversity intactness is urgently needed to further improve the GLOBIO model. We further note that our projections do not account for possible increases in future hunting impacts due to the establishment of new settlements, in absence of a settlement expansion model. Similarly, impacts of future roads might be underestimated because we could not account for the future construction of new roads. Although improvements and increased use intensity of existing roads, as assumed in our projections, typically precede the construction of new roads (Dulac, 2018; Kerali, 2003), future increases in road network length are also expected. Recent projections for 2050 suggested increases in 14%–23% of the global road network, as a function of country‐specific estimates of human population and gross domestic product according to the SSP framework (Meijer et al., 2018). More work is needed to more accurately assess the biodiversity impacts resulting from future hunting pressure as well as road expansion. Further work is also required to enable the GLOBIO model to account for possible synergistic or antagonistic interactions between different pressures, which may lead to larger or smaller pressure contributions than expected based on their individual impacts (Brook, Sodhi, & Bradshaw, 2008; Darling & Cote, 2008). As an example, hunting impacts may be exacerbated by habitat loss and fragmentation, because remaining fragments are more accessible to hunters and isolation may reduce the recolonization from non‐hunted source populations (Peres, 2001).

### 4.3 Regional differences

Our results showed large spatial variation in local biodiversity intactness and projected changes therein. The global MSA pattern for 2015 largely resembles the global pattern of the human footprint index (HFI), which aggregates multiple anthropogenic pressure variables and proxies thereof (Venter et al., 2016). High HFI values as well as low MSA values (i.e. high anthropogenic pressure) are found in Western Europe, the eastern parts of the United States and China, and large parts of India. Remaining relatively intact areas are concentrated in the boreal and tundra biomes, the Sahara, Gobi and Australian deserts, and the most remote tropical forests of the Amazon and Congo Basins. The geographical similarity between HFI and MSA maps, despite some differences in the underlying set of pressures considered, reflects that local pressure variables (land use, human population density, roads, railways, fragmentation) typically co‐occur, with spatial patterns primarily driven by the suitability of land for agriculture (Venter et al., 2016). Our projections revealed that further biodiversity declines are expected in some regions irrespective of the scenario, notably in sub‐Saharan Africa. In contrast, the sustainability scenario projections suggest that losses might be substantially lower or even halted in other regions, for example in North‐East Asia (Table S2), mainly due to considerable decreases in land demand. The differences in pressures and projected biodiversity changes among different world regions point towards the need for a more differentiated approach to improve large‐scale scenario analyses, in particular when it comes to target‐seeking rather than exploratory scenarios (Rosa et al., 2017). Differential targets may be needed depending on the feasibility to reduce anthropogenic drivers and pressures in different contexts. For example, reversing trends of biodiversity loss might be feasible in Europe, where human population is projected to decline (KC & Lutz, 2017). However, targets for sub‐Saharan Africa may need to be different (for example, no or limited further loss) in order to ensure feasibility, given the considerable projected increase in human population (KC & Lutz, 2017) and other sustainable development goals to be attained (UN General Assembly, 2015). Similarly, region‐specific measures could be proposed. For example, measures to reduce food waste could be targeted at final consumers in wealthier regions, while a focus on reducing on‐field post‐harvest losses could be more attainable in sub‐Saharan Africa and South and Southeast Asia (Kok et al., 2018). In addition, impacts need to be quantified for different complementary dimensions of biodiversity, particularly because responses to environmental change may differ among metrics and scales (McGill, Dornelas, Gotelli, & Magurran, 2015; Santini et al., 2017; Schipper, Belmaker, et al., 2016). For example, the MSA metric in GLOBIO does not account for spatial differences in species richness and may therefore miss out on the disproportional impacts in tropical regions in terms of numbers of species lost (Barlow et al., 2018). Similarly, aspects of spatial turnover (beta diversity) are not included in GLOBIO; hence signals of biotic homogenization or heterogenization are not picked up (Socolar, Gilroy, Kunin, & Edwards, 2016). A suite of complementary biodiversity models, combined with scenario settings better tailored to the regional and local context, is needed to further improve scenario‐based biodiversity modelling (Kim et al., 2018; Rosa et al., 2017). Ensemble and probabilistic modelling approaches are recommended to account for model and parameter uncertainties, which were not accounted for in the present study, including the significant uncertainties in underlying climate and land‐use projections (Stehfest et al., 2019; Thuiller, Gueguen, Renaud, Karger, & Zimmermann, 2019). These improvements are urgently needed in order to better inform decision‐making aimed at safeguarding biodiversity.
