# Supplementary material for: Projecting terrestrial biodiversity intactness with GLOBIO 4
Source: Glob Chang Biol. 2019 Nov 3;26(2):760–71. doi: 10.1111/gcb.14848 (PMC7028079; doi:10.1111/gcb.14848)
Supplement: Supplementary file 1 [file GCB-26-760-s001.docx]

**SUPPORTING MATERIAL**

**Projecting terrestrial biodiversity intactness with GLOBIO 4**

Aafke M. Schipper^1,2*^, Jelle P. Hilbers^1^, Johan R. Meijer^1^, Laura H. Antão^3,4^, Ana Benítez-López^2,5^, Melinda M.J. de Jonge^2^, Luuk H. Leemans^2^, Eddy Scheper^6^, Rob Alkemade^1,7^, Jonathan C. Doelman^1^, Sido Mylius^1^, Elke Stehfest^1^, Detlef P. van Vuuren^1,8^, Willem-Jan van Zeist^1^, Mark A.J. Huijbregts^2^

^1^PBL Netherlands Environmental Assessment Agency, The Hague, The Netherlands; ^2^Department of Environmental Science, Institute for Water and Wetland Research, Radboud University, Nijmegen, The Netherlands; ^3^Centre for Biological Diversity, University of St Andrews, Sir Harold Mitchell Building, St Andrews, Scotland UK; ^4^Research Centre for Ecological Change, Organismal and Evolutionary Biology Research Programme, University of Helsinki, Helsinki, Finland; ^5^Integrative Ecology Group, Estación Biológica de Doñana, Sevilla, Spain; ^6^ARIS, Utrecht, The Netherlands; ^7^Environmental Systems Analyses Group, Wageningen University, The Netherlands; ^8^Faculty of Geosciences, Utrecht University, The Netherlands.

^*^ Corresponding author; [Aafke.Schipper@pbl.nl](mailto:Aafke.Schipper@pbl.nl); [A.Schipper@science.ru.nl](mailto:A.Schipper@science.ru.nl)

**Contents**

Text section S1 Pressure-impact relationships

Text section S2 Downscaling land-use data

Figure S1 Locations of data sources for MSA pressure-impact relationships

Figure S2 Coverage of pressure input data by pressure-impact relationships

Figure S3 Global patterns in MSA projected for plants

Figure S4 Global patterns in MSA projected for warm-blooded vertebrates

Figure S5 Contributions of the different pressures to the loss in MSA

Table S1 Impacts accounted for per land-use type

Table S2 Area-weighted mean MSA values per scenario and IPBES region

Table S3 Spatial variability in MSA values per scenario and IPBES region

Table S4 MSA losses per pressure, scenario and IPBES region

Table S5 Global total area per land-use type per scenario

References

Annex S1 Data sources used from the PREDICTS database

**Text section S1 Pressure-impact relationships**

***Impacts of climate change***

To establish pressure-impact relationships for climate change, we used the database recently published by Nunez *et al*. (2019), which contains information on local biodiversity intactness retrieved from 97 bioclimatic envelope modelling studies. From this dataset we selected data on the fraction of remaining species (FRS), which represents the ratio between the number of species remaining after climate change and the original number of species in a location (typically a grid cell in the underlying bioclimatic envelope modelling study). Thus, FRS is a relative index between 0 (no original species present) and 1 (all original species present), which we considered a proxy for MSA. Per study, region and climate scenario, FRS values were provided as average across the locations within a region (Nunez *et al.*, 2019). In total, 135 FRS values from 31 studies were available for plants and 141 FRS values from 19 studies for warm-blooded vertebrates (Figure S1). Because the underlying studies differed in the climatic variables selected, we used the global mean temperature increase corresponding with each climate scenario as common explanatory variable, following Nunez *et al.* (2019). Per taxonomic group, we established a mixed beta regression model to relate FRS to the global mean temperature increase (^o^C), using study as random intercept. Because responses to climate change are expected to differ between tropical, temperate and Arctic regions (Deutsch *et al.*, 2008, Post *et al.*, 2009), we tested for an interaction with climate zone. To that end, we assigned each observation to either the tropics or to non-tropics (because of a lack of data to distinguish Arctic regions separately), delineating the tropics as the biomes 'Tropical and subtropical moist broadleaf forests', 'Tropical and subtropical dry broadleaf forests', 'Tropical and subtropical coniferous forests', and 'Tropical and subtropical grasslands, savannas, and shrublands', as distinguished by Dinerstein et al. (2017). However, the overall model had a lower BIC value.

***Impacts of atmospheric nitrogen deposition***

We retrieved a pressure-impact relationship for nitrogen deposition (kg∙ha∙yr^-1^) based on a data from field experiments that quantified responses of natural and semi-natural terrestrial plant communities to nitrogen addition. We used the database published by Midolo *et al*. (2019), which contains observations from 115 nitrogen addition experiments reported in 85 studies. Of these, 37 studies reported ratios of individual species abundance at a given level of N addition relative to the abundance in a control plot, which we used to calculate 89 MSA values (Figure S1). We used these MSA values as input to a mixed beta regression model with dataset within study as nested random intercept and the log_10_-transformed amount of nitrogen addition (kg∙ha∙yr^-1^) as fixed effect. We tested for interactions with mean annual temperature, following Midolo *et al*. (2019). However, the model including only nitrogen addition had the lowest BIC value.

***Impacts of land use***

We quantified relationships between MSA and land use based on monitoring data from the 2016 release of the PREDICTS database (<https://data.nhm.ac.uk/dataset/the-2016-release-of-the-predicts-database>), which contains spatial comparisons of species’ assemblages in particular land-use types and intensity levels (Hudson *et al.*, 2017). PREDICTS categorizes each record into one of nine predominant land-use (LU) types: ‘Primary vegetation’, ‘Mature secondary vegetation’, ‘Intermediate secondary vegetation’, ‘Young secondary vegetation’, ‘Secondary vegetation of indeterminate age’, ‘Plantation forest’, ‘Cropland’, ‘Pasture’, or ‘Urban’. Furthermore, each record falls into one of four land-use intensities (UI): ‘Minimal use’, ‘Light use’, ‘Intense use’, and ‘Cannot decide’. Details on the land-use classification are provided by Hudson *et al.* (2014). We extracted observations from studies that reported abundances of plant and warm-blooded vertebrate species in ‘Primary vegetation – minimal use’, which we used as controls, and at least one other land use type. We then assigned each record from the selected studies a GLOBIO land-use category (‘Secondary vegetation’, ‘Plantation’, ‘Cropland - Minimal use’, ‘Cropland - Intense use’, ‘Pasture - Minimal use’, ‘Pasture - Intense use’ and ‘Urban’). We excluded observations from PREDICTS land-use categories that we could not match with a GLOBIO category. An overview of studies used from PREDICTS is provided in Annex S1.

Typically, multiple sites were sampled in a given LU category within a dataset. We averaged the abundance records per species, dataset and LU category over the sample sites. We then calculated for each species within a LU type within a dataset an abundance ratio by dividing its abundance in the disturbed habitat by its abundance in the reference site, and we retrieved MSA values by averaging the truncated abundance ratios per LU type, species group, and dataset. This resulted in 55 MSA values from 32 studies for plants and 85 MSA values from 48 studies for warm-blooded vertebrates (Figure S1), which we used as input to mixed beta regression models with dataset within study as nested random intercept and the GLOBIO land-use classes as fixed effects.

***Impacts of habitat fragmentation***

To quantify the impact relationship for habitat fragmentation, we used data from the PREDICTs database (i.e., the same database as used for quantifying the land-use impacts). Because fragmentation impacts in GLOBIO are confined to warm-blooded vertebrates and natural vegetation, we selected from PREDICTS observations for birds and mammals from the categories "Primary vegetation – minimal use" and "Mature secondary vegetation – minimal use" with quantitative information available on the patch size. Some patches had size denoted as ‘-1’, meaning that the patch was so large it was not measured (Hudson *et al.*, 2014). To these patches we assigned a size slightly larger than the largest value reported in PREDICTS. We then selected datasets that included multiple patches with at least one patch of 10,000 ha or larger in size and used the largest patch as undisturbed reference (control). Thus, we assumed that fragmentation impacts are absent in patches of 10,000 ha or more, due to a shortage of datasets including larger patches. We then calculated per species, patch and dataset an abundance ratio by dividing its abundance in the smaller patch by its abundance in the control patch and obtained MSA values by averaging the truncated abundance ratios per patch size and dataset, which yielded 39 MSA values from seven studies (Figure S1). We used these MSA values as input to mixed beta regression models with dataset nested in study as random intercept and the log_10_-transformed patch size (ha) as fixed effect.

***Impacts of road disturbance***

In GLOBIO, impacts of road disturbance are quantified based on a relationship between MSA and the distance to the closest road (m). To quantify the relationship we used the database collected by Benítez-López *et al.* (2010) as a starting point and extended this with additional observations from more recent literature. The studies included in the database focused on the impacts of road construction and use on wildlife populations in the areas adjacent to roads. Noise, movements and exhausts of cars, for example, disturb habitat adjacent to roads and create road-effect zones that extend beyond the road itself. Papers addressing secondary impacts of road development (i.e. human encroachment and hunting) were discarded from the database, thereby precluding any overlap with studies included in the hunting database (see below). Per dataset within the database, we calculated MSA values based on the abundance of species measured at a given distance or within a distance interval from the road relative to their abundance at a larger control distance where road impacts were presumably absent. The extended database included 204 MSA values from 34 studies (Figure S1), which we used as input to mixed beta regression models with dataset within study as nested random intercept and the log_10_-transformed distance to the road (m) as fixed effect.

***Impacts of hunting***

In GLOBIO, impacts of hunting in the tropics are quantified based on the distance to hunters’ access points (i.e., small settlements) within tropical biomes. Based on the central-place foraging hypothesis, hunting intensity is generally higher in the proximity of hunters’ access points (Abernethy *et al.*, 2013, Benítez-López *et al.*, 2017), generating gradients of increasing species densities up to a distance where no effect is observed (i.e., species depletion distances). Although a proxy, distance to a settlement has been identified as the main factor explaining animal abundance declines due to hunting pressure, even after accounting for other factors such as human population density, poverty levels or accessibility to urban markets (Benítez-López *et al.*, 2019). We quantified the relationship between MSA and distance to settlement based on a database with mammal and bird abundances measured in hunted versus unhunted sites (Benítez-López *et al.*, 2017, Benítez-López *et al.*, 2019). For this database, only studies that assessed the impact of hunting on wildlife abundance were included. Specifically, the studies had to report species abundance in at least one hunted area and one unhunted control area, and at increasing distance from access points. Studies with potential confounding effects due to other disturbances (e.g., hunted and logged area versus unhunted unlogged area) were discarded. Also, we did not include studies that reported hunting on managed wild populations (i.e., with release of farm-reared individuals or subjected to culling) and/or populations used for recreational or trophy hunting. As MSA is supposed to represent overall assemblage-level intactness, we included observations of species irrespective of whether they are hunted or not. Overall, approximately 20% of the mammal species in our dataset have a body mass < 1 kg, hence are usually not hunted (Ripple *et al.*, 2016). As for birds, 38% of the species in the dataset belong to families that are usually not hunted (Redford, 1992).

Per dataset within the database, we calculated MSA values based on the abundance of species measured at a given distance or within a distance interval from an access point relative to their abundance at a larger control distance where hunting impacts were presumably absent. This resulted in 465 MSA values from 125 studies (Figure S1) that we used as input to mixed beta regression models with dataset within study as nested random intercept, country as crossed random intercept (to account for possible cultural differences in hunting; Benítez-López *et al.* 2019) and the log_10_-transformed distance to hunters' access point (km) as fixed effect. Following Benítez-López *et al.* (2019), we tested for a possible effect of human population density, using log_10_-transformed population density values from the Gridded Population of the World dataset (CIESIN, 2017) matched as closely as possible to the location and year of the study. However, the model with distance to settlements as only predictor had a lower BIC value.

**Text section S2 Downscaling land-use data**

***Suitability layers***

To compile the downscaled land-use maps, we first established a suitability layer for each of four major land-use types (urban area, cropland, pasture and forestry). Because spatial clustering and edge expansion have been identified as key processes in the growth of urban areas and croplands (Ay *et al.*, 2017, Huang *et al.*, 2019, Richards, 2018), we retrieved the suitability layers for urban area and cropland based on the proximity to existing urban area and croplands. To that end, we calculated the Euclidean distance to urban area (class 190) or croplands (classes 10-40) in the recently published ESA-CCI map for 2015 (ESA, 2017)^a^, assigned the highest suitability to existing cropland or urban area, and inverted and normalized the distances to existing urban area or cropland. We further set the suitability of non-urban and non-cropland cells within protected areas to zero, based on the assumption that within protected areas there would be no expansion of urban and cropland areas beyond those present in 2015. Protected areas were delineated based on the World Database of Protected Areas (WDPA^b^).

For pasture, we used a suitability layer based on the density of ruminant livestock species (cattle, goat and sheep), which we retrieved from FAO’s gridded livestock of the world dataset (GLW; head per km^2^, 30 arc-seconds) (Robinson *et al.*, 2014). The GLW provides modelled livestock densities based on detailed subnational livestock statistics combined with a set of predictor variables related to climate, vegetation, topography and demography. We converted the livestock species' densities to tropical livestock units to correct for differences in body mass among livestock species (Petz *et al.*, 2014), summed the units per grid and normalized to achieve suitability values ranging from 0 to 1. Protected areas automatically got zero suitability because the GLW map layers assume that there is no livestock in protected areas (Robinson *et al.*, 2014).

To establish the suitability layer for forestry, we assumed that accessibility to wood is primarily determined by elevation, proximity to infrastructure, and the presence of protected areas (FAO, 2000). We calculated the Euclidean distance to the nearest road or, in South-America, the nearest road or river (FAO, 2000). We retrieved a global roads map from the GRIP database (Meijer *et al.*, 2018) and delineated the rivers using the Digital Chart of the World^c^ and the Global Lakes and Wetland Database^d^). We inverted and normalized the distances and multiplied the resulting values with inverted and normalized elevation values (retrieved from NASA Shuttle Radar Topographic Mission (SRTM) data^e^, which we resampled to 10 arc-seconds) to arrive at suitability values between 0 and 1. Additionally, we assumed that there would be no forestry activities in protected areas and therefore set the suitability values for forestry within protected areas to zero. Finally, we clipped the forestry suitability layer to land cover with trees, using the ESA CCI land cover map for 2015 (classes 50-110), and set the suitability of other cells to zero.

***Present-day land-use map***

To establish a land-use map for the reference year (2015), we applied the land-use allocation routine using the ESA CCI land-cover map for 2015 as 'background' map, the suitability layers as described above, and country-level total areas (i.e., ‘claims’) of urban, cropland, pasture and forestry land. To obtain claims as representative as possible for 2015, we used two data sources: we retrieved the claims for urban area and cropland from the ESA CCI map for 2015 (i.e., the land-cover background map itself) and claims for pasture and forestry (which cannot be distinguished from natural grasslands or natural forest, respectively, based on remotely sensed land-cover maps) from country-level statistics for 2015 as assembled by the FAO^f^. We defined the pasture claim as the sum of the country-level totals of permanent and temporary meadows and the forestry claim as the country-level total of planted forest. Using the same source for both the suitability layers and the claims of urban and cropland areas ensured that the ‘claims’ for urban area and cropland were assigned only to cells identified as urban or cropland in 2015, i.e., the allocated layers were identical to the original ESA map for 2015. Thus, we stayed as closely as possible to urban and cropland claims and patterns as observed in 2015.

***Future land-use maps***

To obtain the future land-use maps, we first calculated country-level total areas of the four land use types (urban, cropland, pasture and forestry) for each scenario-year (i.e., 2015 and three times 2050, for each SSP scenario) from the LUH2 dataset (Hurtt *et al.*, in prep.) (<http://luh.umd.edu/data.shtml>). The LUH2 dataset contains harmonized land-use data from different integrated assessment models (IAMS) that provided so-called 'marker' projections, i.e., projections that are considered the most representative of each SSP (Riahi *et al.*, 2017): projections from IMAGE for SSP1, from AIM for SSP3, and data from REMIND-MAgPIE for SSP5 (Popp *et al.*, 2017). We calculated the cropland claims as the sum of the areas of the five cropland types included in LUH2 (c3ann + c3per + c4ann + c4per + c3nfx), the pasture claim as the sum of the areas of pasture and rangeland, and the forestry claim as the sum of the wood harvest from forested cells and non-forested cells with primary vegetation (primf_harv + primn_harv). We then calculated for each future scenario year the change in area of each land-use type relative to the area in 2015 and added this difference to the claims defined for 2015 (as described above), taking the sum as the total claim. Thus, we used the LUH2 data to define the change in claims rather than the claims themselves, reasoning that temporal trends in land use are well represented by the integrated assessment models underlying LUH2, but that the present-day situation is better represented by remote sensing data and national statistics as included in our initial land-use map.

***Land-use intensity levels***

After the land allocation we assigned land-use intensity levels to cropland and pasture by overlaying the downscaled land-use maps with the year- and scenario-specific amount of N application as retrieved from the LUH2 data. To that end, we first create total fertilizer maps based on the amount of N application (kg∙ha^-1^) as area-weighted average over the five crop types. We then classified cropland and pasture intensity per cell as light use for N application rates up to and including 100 kg N-input/ha and intense use for rates > 100 kg N-input/ha (Temme & Verburg, 2011).

***Data sources***

^a^ <https://maps.elie.ucl.ac.be/CCI/viewer/>

^b^ <https://www.iucn.org/theme/protected-areas/our-work/quality-and-effectiveness/world-database-protected-areas-wdpa>

^c^ <https://worldmap.harvard.edu/data/geonode:Digital_Chart_of_the_World>

^d^ <https://www.worldwildlife.org/pages/global-lakes-and-wetlands-database>

^e^ <http://srtm.csi.cgiar.org/>

^f^ FAO, 2018. FAOSTAT Land domain. <http://www.fao.org/faostat/en/#data/RL>

**
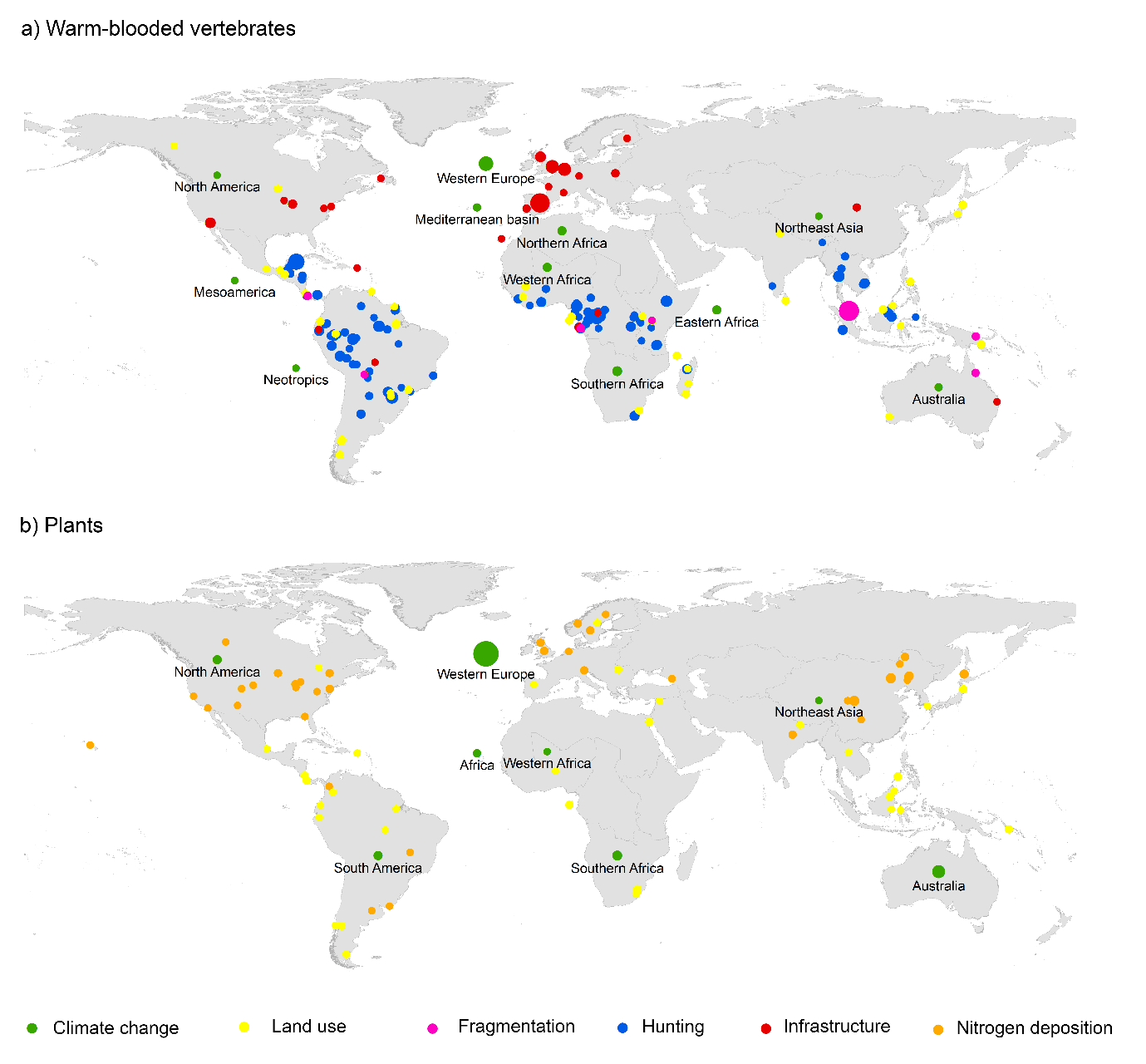
**

**Figure S1** Locations of studies used to retrieve data for establishing the pressure-impact relationships for MSA for a) warm-blooded vertebrates and b) plants. Point size is proportional to unique combinations of dataset and pressure level. Climate change studies (typically covering large areas) were assigned to IPBES sub-regions unless the study encompassed multiple regions (e.g., Neotropics).


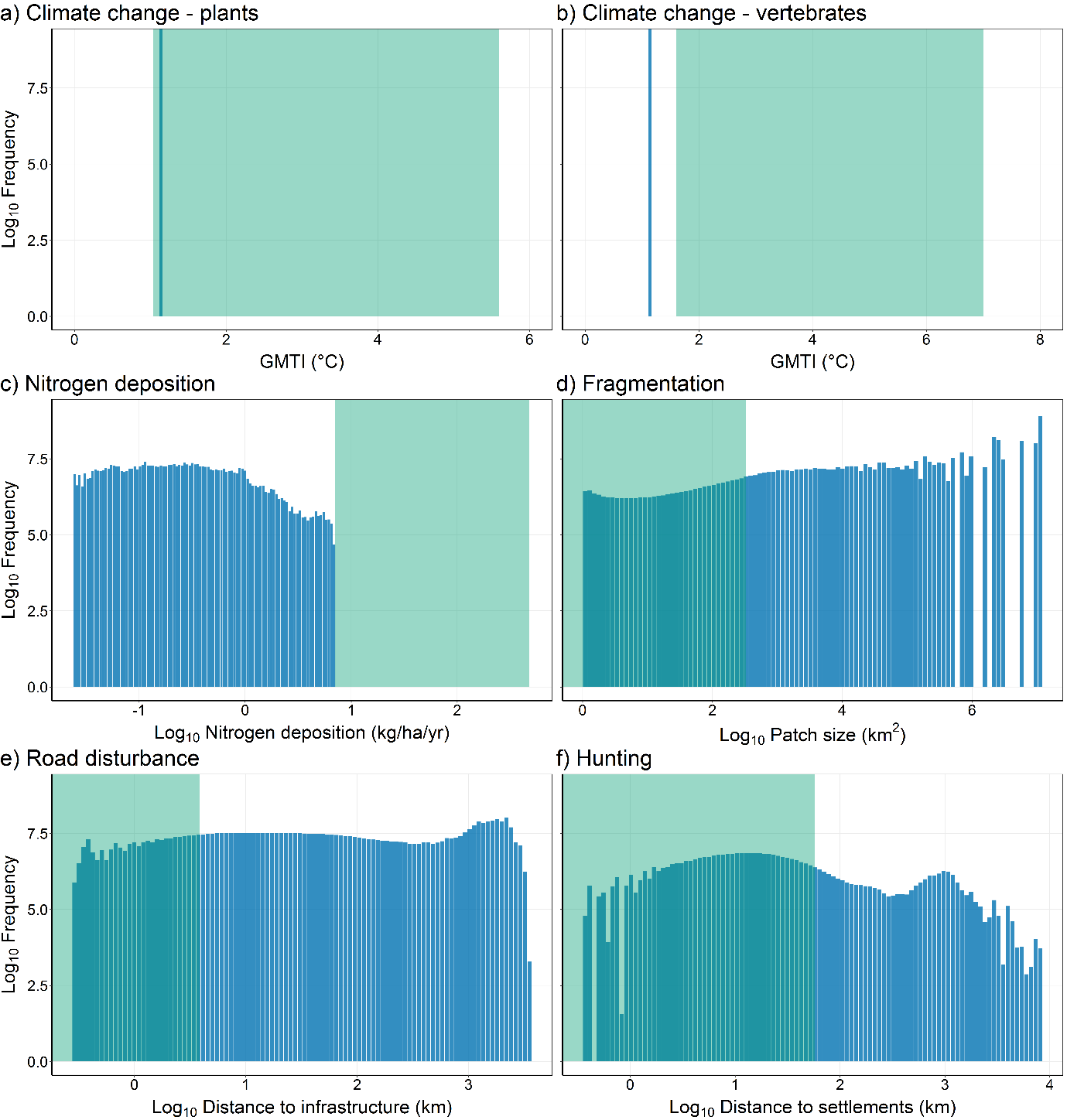


**Figure S2** Distributions of pressure values (2015 situation; blue) compared to the range of input data used to establish the pressure-impact relationships for MSA (green).


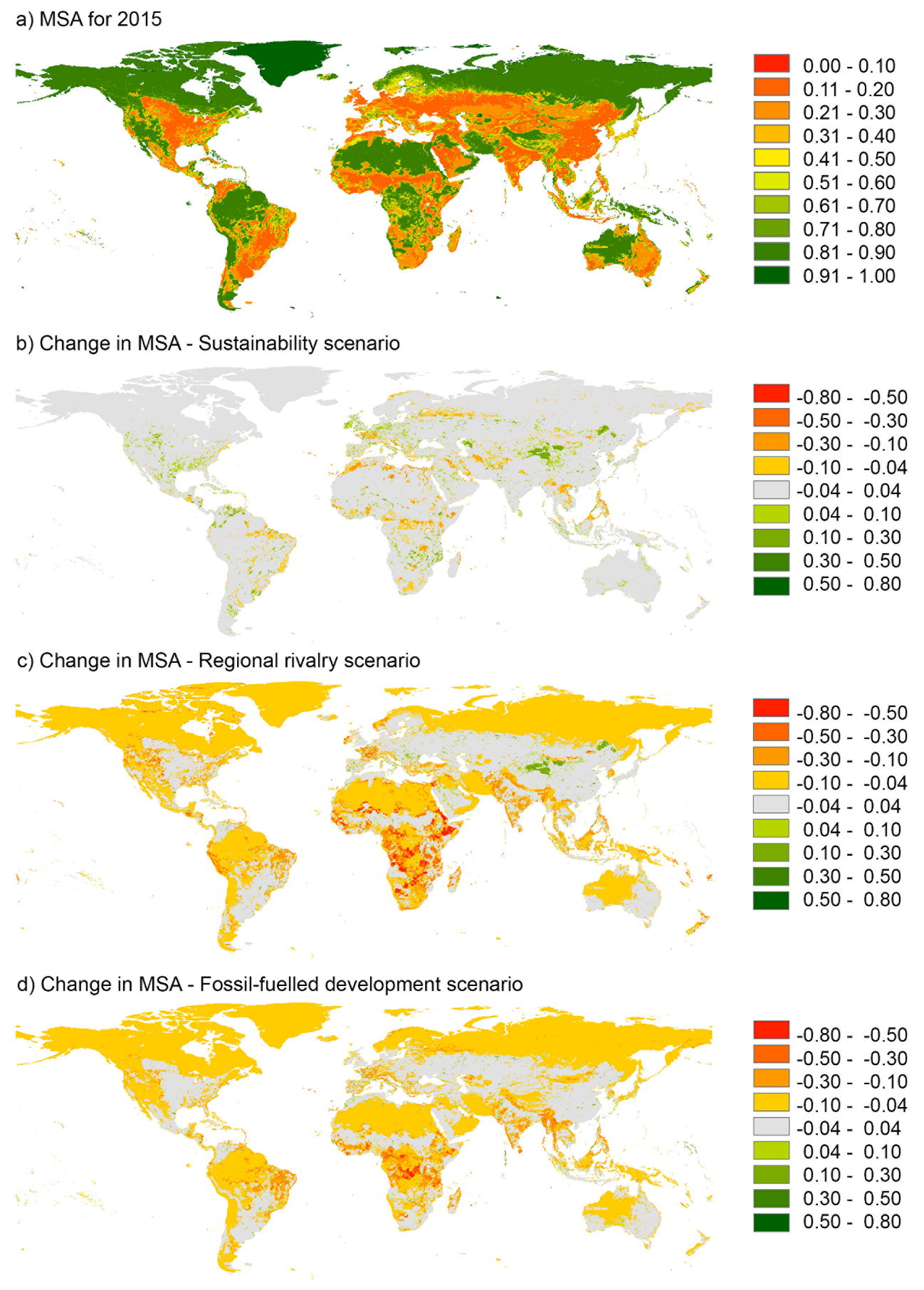


**Figure S3** Global patterns in a) MSA values for 2015 and changes in MSA values from 2015 to 2050 for b) sustainability scenario (SSP1xRCP2.6), c) regional rivalry scenario (SSP3xRCP6.0) and d) fossil-fuelled development scenario (SSP5xRCP8.5) for plants. For visualisation purposes, the maps were resampled to a resolution of 0.25 degree based on mean values.


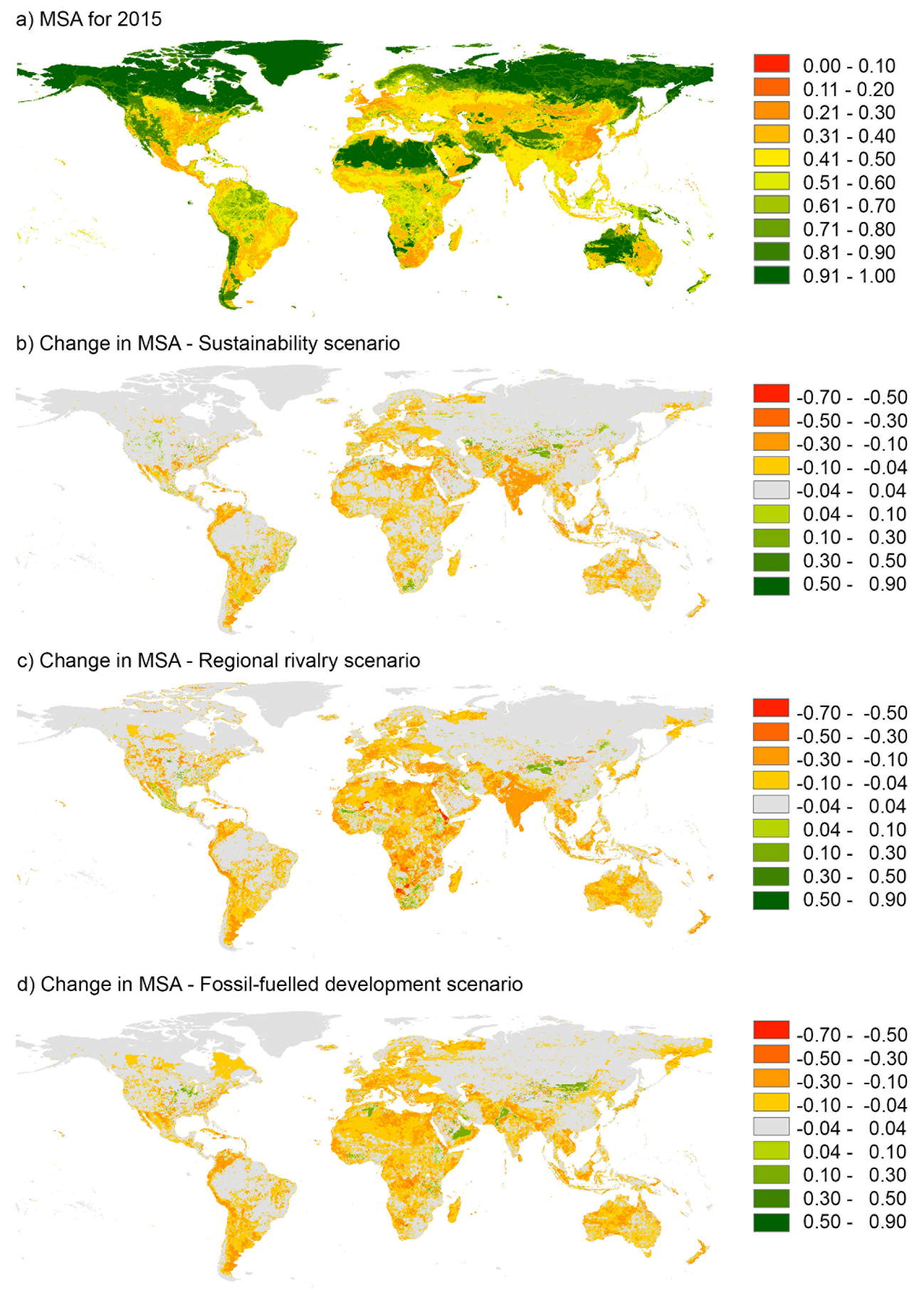


**Figure S4** Global patterns in a) MSA values for 2015 and changes in MSA values from 2015 to 2050 for b) sustainability scenario (SSP1xRCP2.6), c) regional rivalry scenario (SSP3xRCP6.0) and d) fossil-fuelled development scenario (SSP5xRCP8.5) for warm-blooded vertebrates. For visualisation purposes, the maps were resampled to a resolution of 0.25 degree based on mean values.

**
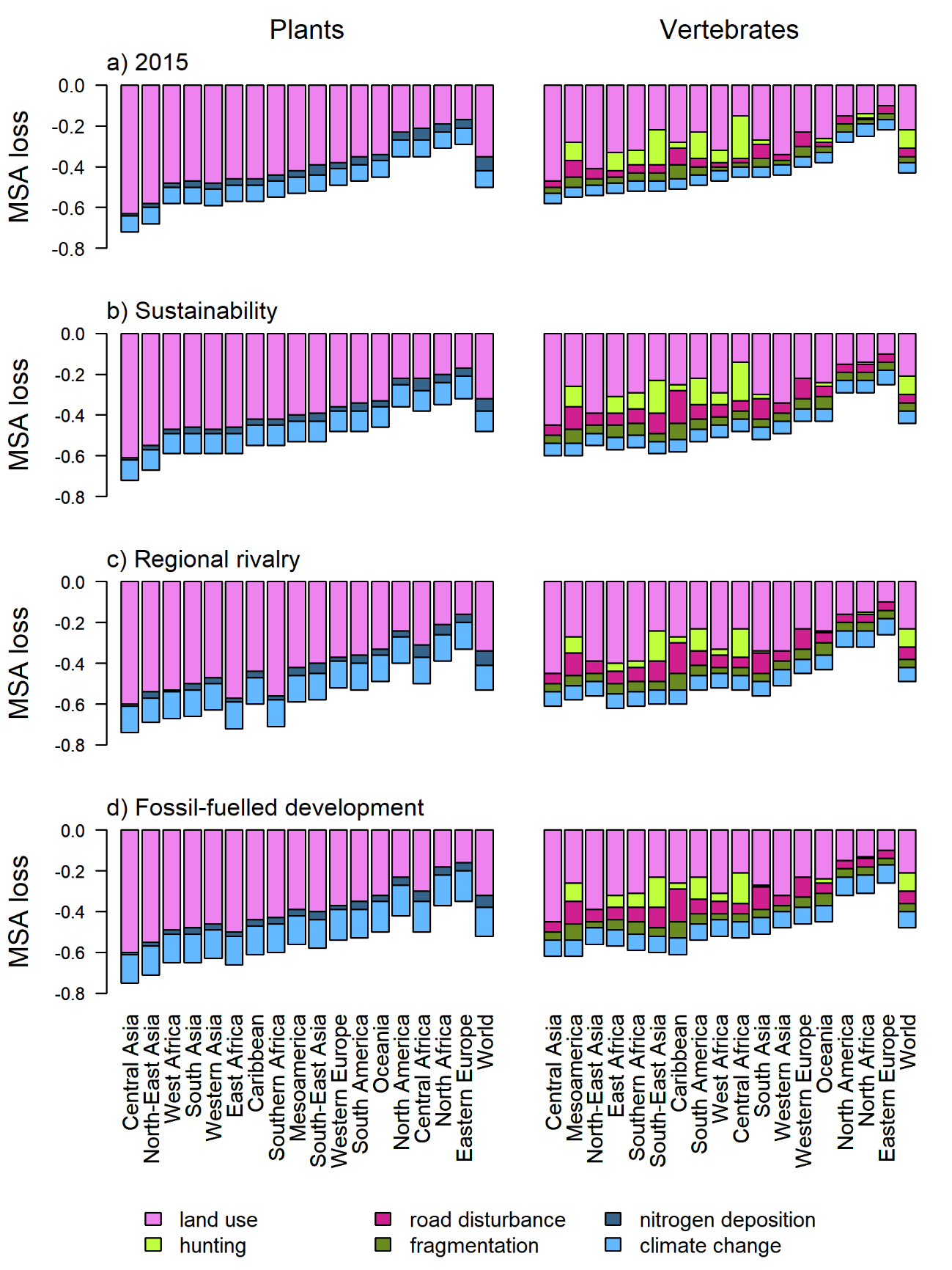
**

**Figure S5** Losses in MSA per pressure, per IPBES sub-region and globally for plants (left) and warm-blooded vertebrates (right) for 2015 (a) and each of the three future scenarios in 2050 (b-d). Underlying numbers are provided in Table S4.

**Table S1** Overview of impacts accounted for per land-use type. 'yes' indicates that the impact is included within the land-use type of concern; 'no' indicates that the impact is not accounted for as the impact of the land use itself is assumed to dominate.

| **Land use** | **Climate**  **change** | **Nitrogen**  **deposition** | **Fragmentation** | **Road**  **disturbance** | **Hunting** |
| --- | --- | --- | --- | --- | --- |
| Urban | yes | no | no | no | no |
| Cropland – Intense use | yes | no | no | yes | no |
| Cropland – Light use | yes | no | no | yes | no |
| Pasture – Intense use | yes | no | no | yes | no |
| Pasture – Light use | yes | no | yes | yes | no |
| Forestry | yes | yes | yes | yes | no |
| Secondary vegetation | yes | yes | yes | yes | yes |
| Primary vegetation | yes | yes | yes | yes | yes |

**Table S2** Area-weighted mean MSA values for 2015 and changes from 2015 to 2050 per scenario, IPBES region and globally. S = sustainability scenario (SSP1xRCP2.6), RR = regional rivalry scenario (SSP3xRCP6.0) and FD = fossil-fuelled development scenario (SSP5xRCP8.5).

| **IPBES region** | **All** | | | | **Plants** | | | | **Vertebrates** | | | |
| --- | --- | --- | --- | --- | --- | --- | --- | --- | --- | --- | --- | --- |
|  | *MSA* | *Δ MSA* | | | *MSA* | *Δ MSA* | | | *MSA* | *Δ MSA* | |  |
|  | 2015 | S | RR | FD | 2015 | S | RR | FD | 2015 | S | RR | FD |
| Central Africa | 0.60 | -0.04 | -0.12 | -0.11 | 0.65 | -0.03 | -0.14 | -0.14 | 0.55 | -0.04 | -0.09 | -0.08 |
| East Africa | 0.45 | -0.03 | -0.12 | -0.07 | 0.43 | -0.02 | -0.14 | -0.09 | 0.47 | -0.04 | -0.10 | -0.05 |
| North Africa | 0.72 | -0.05 | -0.07 | -0.06 | 0.68 | -0.03 | -0.07 | -0.05 | 0.76 | -0.06 | -0.08 | -0.07 |
| Southern Africa | 0.46 | -0.02 | -0.12 | -0.05 | 0.45 | -0.01 | -0.15 | -0.05 | 0.48 | -0.04 | -0.08 | -0.05 |
| West Africa | 0.47 | -0.03 | -0.07 | -0.06 | 0.41 | -0.01 | -0.08 | -0.06 | 0.53 | -0.04 | -0.06 | -0.05 |
| Caribbean | 0.46 | -0.03 | -0.06 | -0.06 | 0.43 | 0.01 | -0.03 | -0.04 | 0.49 | -0.07 | -0.09 | -0.09 |
| Mesoamerica | 0.46 | -0.02 | -0.04 | -0.05 | 0.47 | 0.00 | -0.05 | -0.04 | 0.45 | -0.04 | -0.03 | -0.06 |
| North America | 0.68 | -0.01 | -0.04 | -0.04 | 0.64 | 0.00 | -0.05 | -0.05 | 0.72 | -0.01 | -0.03 | -0.04 |
| South America | 0.52 | -0.03 | -0.05 | -0.06 | 0.53 | -0.01 | -0.05 | -0.06 | 0.52 | -0.04 | -0.05 | -0.05 |
| North-East Asia | 0.38 | 0.00 | -0.01 | -0.02 | 0.31 | 0.01 | 0.00 | -0.02 | 0.45 | -0.01 | -0.02 | -0.02 |
| Oceania | 0.58 | -0.03 | -0.05 | -0.05 | 0.55 | -0.01 | -0.04 | -0.04 | 0.62 | -0.05 | -0.07 | -0.07 |
| South-East Asia | 0.48 | -0.04 | -0.07 | -0.07 | 0.48 | -0.02 | -0.06 | -0.07 | 0.48 | -0.07 | -0.08 | -0.07 |
| South Asia | 0.49 | -0.05 | -0.10 | -0.06 | 0.42 | -0.01 | -0.07 | -0.07 | 0.55 | -0.09 | -0.12 | -0.06 |
| Western Asia | 0.48 | -0.03 | -0.05 | -0.04 | 0.42 | -0.01 | -0.04 | -0.04 | 0.55 | -0.05 | -0.06 | -0.04 |
| Western Europe | 0.56 | -0.02 | -0.05 | -0.06 | 0.52 | 0.00 | -0.04 | -0.05 | 0.60 | -0.04 | -0.06 | -0.06 |
| Central Asia | 0.35 | -0.01 | -0.02 | -0.03 | 0.28 | 0.00 | -0.01 | -0.02 | 0.42 | -0.01 | -0.03 | -0.04 |
| Eastern Europe | 0.74 | -0.02 | -0.03 | -0.05 | 0.70 | -0.01 | -0.04 | -0.06 | 0.78 | -0.02 | -0.03 | -0.04 |
| World | 0.56 | -0.02 | -0.06 | -0.05 | 0.53 | -0.01 | -0.06 | -0.06 | 0.60 | -0.04 | -0.05 | -0.05 |

**Table S3** Spatial variability in MSA (2015) and in changes in MSA (from 2015 to 2050) per IPBES region, expressed as 5 - 95 percentiles across the grid cells. S = sustainability scenario (SSP1xRCP2.6), RR = regional rivalry scenario (SSP3xRCP6.0), FD = fossil-fuelled development scenario (SSP5xRCP8.5).

|  | **All** |  |  |  | **Plants** |  |  |  | **Vertebrates** |  |  |  |
| --- | --- | --- | --- | --- | --- | --- | --- | --- | --- | --- | --- | --- |
|  | **MSA** |  |  |  | **MSA** |  |  |  | **MSA** |  |  |  |
|  | 2015 | S | RR | FD | 2015 | S | RR | FD | 2015 | S | RR | FD |
| Central Africa | 0.27 - 0.9 | -0.07 - 0 | -0.46 - -0.01 | -0.45 - -0.01 | 0.12 - 0.87 | -0.03 - 0 | -0.61 - -0.01 | -0.61 - 0 | 0.32 - 0.93 | -0.11 - 0 | -0.33 - 0 | -0.3 - 0 |
| East Africa | 0.26 - 0.86 | -0.05 - 0 | -0.51 - 0.03 | -0.44 - 0.03 | 0.12 - 0.85 | -0.03 - 0 | -0.63 - 0.03 | -0.12 - 0 | 0.28 - 0.88 | -0.09 - 0 | -0.32 - 0 | -0.14 - 0 |
| North Africa | 0.27 - 0.9 | -0.09 - 0 | -0.13 - -0.01 | -0.11 - -0.01 | 0.12 - 0.87 | -0.02 - 0 | -0.06 - -0.01 | -0.07 - 0 | 0.31 - 0.93 | -0.16 - 0 | -0.18 - 0 | -0.17 - 0 |
| Southern Africa | 0.24 - 0.88 | -0.05 - 0.04 | -0.53 - 0.04 | -0.15 - 0.02 | 0.12 - 0.86 | -0.02 - 0 | -0.63 - 0.04 | -0.07 - 0 | 0.26 - 0.91 | -0.1 - 0 | -0.37 - 0.15 | -0.12 - 0 |
| West Africa | 0.26 - 0.9 | -0.06 - 0 | -0.44 - 0.03 | -0.25 - 0.02 | 0.12 - 0.87 | -0.02 - 0 | -0.61 - 0.03 | -0.12 - 0 | 0.29 - 0.93 | -0.17 - 0 | -0.27 - 0.15 | -0.22 - 0.14 |
| Caribbean | 0.22 - 0.82 | 0 - 0 | -0.25 - 0.03 | -0.25 - 0 | 0.12 - 0.86 | 0 - 0 | -0.01 - 0.03 | -0.01 - 0 | 0.16 - 0.79 | 0 - 0 | -0.01 - 0 | -0.02 - 0 |
| Mesoamerica | 0.21 - 0.86 | -0.05 - 0 | -0.17 - 0.05 | -0.14 - 0 | 0.12 - 0.85 | -0.02 - 0 | -0.07 - 0.05 | -0.07 - 0 | 0 - 0.88 | -0.1 - 0 | -0.16 - 0.13 | -0.1 - 0 |
| North America | 0.24 - 0.9 | -0.02 - 0 | -0.07 - -0.01 | -0.06 - -0.01 | 0.12 - 0.89 | -0.02 - 0 | -0.05 - -0.01 | -0.07 - 0 | 0.28 - 0.93 | -0.02 - 0 | -0.03 - 0 | -0.04 - 0 |
| South America | 0.24 - 0.87 | -0.09 - 0.03 | -0.24 - -0.01 | -0.21 - 0.02 | 0.12 - 0.86 | -0.02 - 0 | -0.06 - -0.01 | -0.07 - 0 | 0.27 - 0.88 | -0.17 - 0 | -0.15 - 0 | -0.18 - 0 |
| North-East Asia | 0.21 - 0.88 | -0.05 - 0.07 | -0.06 - 0.19 | -0.1 - 0.03 | 0.12 - 0.85 | -0.01 - 0.05 | -0.05 - 0.19 | -0.07 - 0 | 0.28 - 0.91 | -0.15 - 0.1 | -0.12 - 0.1 | -0.12 - 0.13 |
| Oceania | 0.26 - 0.9 | -0.03 - 0 | -0.11 - -0.01 | -0.11 - -0.01 | 0.12 - 0.87 | -0.02 - 0 | -0.05 - -0.01 | -0.07 - 0 | 0.29 - 0.92 | -0.05 - 0 | -0.06 - 0 | -0.07 - 0 |
| South-East Asia | 0.22 - 0.74 | -0.06 - 0 | -0.31 - 0 | -0.33 - 0.02 | 0.12 - 0.86 | -0.02 - 0 | -0.06 - 0 | -0.06 - 0 | 0.28 - 0.64 | -0.13 - 0 | -0.17 - 0 | -0.09 - 0 |
| South Asia | 0.22 - 0.88 | -0.1 - 0 | -0.43 - 0.04 | -0.34 - 0.07 | 0.12 - 0.86 | -0.02 - 0 | -0.12 - 0.04 | -0.07 - 0 | 0.25 - 0.91 | -0.21 - 0 | -0.25 - 0 | -0.19 - 0.14 |
| Western Asia | 0.26 - 0.88 | -0.08 - 0 | -0.11 - -0.01 | -0.1 - 0.03 | 0.12 - 0.85 | -0.02 - 0 | -0.07 - -0.01 | -0.07 - 0 | 0.3 - 0.92 | -0.17 - 0 | -0.18 - 0 | -0.17 - 0.13 |
| Western Europe | 0.21 - 0.92 | -0.03 - 0 | -0.15 - 0 | -0.14 - 0 | 0.12 - 0.91 | -0.02 - 0 | -0.05 - 0 | -0.07 - 0 | 0.26 - 0.93 | -0.05 - 0 | -0.08 - 0 | -0.07 - 0 |
| Central Asia | 0.25 - 0.88 | -0.08 - 0.04 | -0.06 - -0.01 | -0.06 - -0.01 | 0.12 - 0.85 | -0.06 - 0 | -0.06 - -0.01 | -0.06 - 0 | 0.27 - 0.9 | -0.17 - 0.15 | -0.09 - 0 | -0.1 - 0 |
| Eastern Europe | 0.27 - 0.9 | -0.02 - 0 | -0.05 - -0.01 | -0.06 - -0.01 | 0.12 - 0.88 | -0.02 - 0 | -0.05 - -0.01 | -0.07 - 0 | 0.3 - 0.93 | -0.03 - 0 | -0.04 - 0 | -0.05 - 0 |

**Table S4** MSA losses per pressure, scenario and IPBES region. CC = climate change, ND = nitrogen deposition, LU = land use, F = fragmentation, RD = road disturbance, H = hunting; S = sustainability scenario (SSP1xRCP2.6), RR = regional rivalry scenario (SSP3xRCP6.0), FD = fossil-fuelled development scenario (SSP5xRCP8.5).

| **Group** | **Region** | **Scenario** | **Pressure** | | | | | | |
| --- | --- | --- | --- | --- | --- | --- | --- | --- | --- |
|  |  |  | **CC** | **ND** | **LU** | **F** | **RD** | **H** | **Total** |
| Plants | Caribbean | 2015 | -0.08 | -0.03 | -0.46 | 0 | 0 | 0 | -0.57 |
|  |  | S | -0.10 | -0.03 | -0.42 | 0 | 0 | 0 | -0.56 |
|  |  | RR | -0.13 | -0.03 | -0.44 | 0 | 0 | 0 | -0.60 |
|  |  | FD | -0.14 | -0.03 | -0.44 | 0 | 0 | 0 | -0.61 |
|  | Central Africa | 2015 | -0.08 | -0.06 | -0.21 | 0 | 0 | 0 | -0.35 |
|  |  | S | -0.10 | -0.06 | -0.22 | 0 | 0 | 0 | -0.38 |
|  |  | RR | -0.13 | -0.06 | -0.31 | 0 | 0 | 0 | -0.50 |
|  |  | FD | -0.15 | -0.05 | -0.30 | 0 | 0 | 0 | -0.49 |
|  | Central Asia | 2015 | -0.08 | -0.01 | -0.63 | 0 | 0 | 0 | -0.72 |
|  |  | S | -0.10 | -0.01 | -0.61 | 0 | 0 | 0 | -0.72 |
|  |  | RR | -0.13 | -0.01 | -0.60 | 0 | 0 | 0 | -0.73 |
|  |  | FD | -0.14 | -0.01 | -0.60 | 0 | 0 | 0 | -0.74 |
|  | East Africa | 2015 | -0.08 | -0.03 | -0.46 | 0 | 0 | 0 | -0.57 |
|  |  | S | -0.10 | -0.03 | -0.46 | 0 | 0 | 0 | -0.59 |
|  |  | RR | -0.13 | -0.02 | -0.57 | 0 | 0 | 0 | -0.72 |
|  |  | FD | -0.14 | -0.02 | -0.50 | 0 | 0 | 0 | -0.66 |
|  | Eastern Europe | 2015 | -0.08 | -0.04 | -0.17 | 0 | 0 | 0 | -0.30 |
|  |  | S | -0.11 | -0.04 | -0.17 | 0 | 0 | 0 | -0.31 |
|  |  | RR | -0.13 | -0.04 | -0.16 | 0 | 0 | 0 | -0.34 |
|  |  | FD | -0.15 | -0.04 | -0.16 | 0 | 0 | 0 | -0.35 |
|  | Mesoamerica | 2015 | -0.08 | -0.03 | -0.42 | 0 | 0 | 0 | -0.53 |
|  |  | S | -0.10 | -0.03 | -0.40 | 0 | 0 | 0 | -0.53 |
|  |  | RR | -0.13 | -0.04 | -0.42 | 0 | 0 | 0 | -0.58 |
|  |  | FD | -0.14 | -0.03 | -0.39 | 0 | 0 | 0 | -0.57 |
|  | North Africa | 2015 | -0.08 | -0.04 | -0.19 | 0 | 0 | 0 | -0.32 |
|  |  | S | -0.11 | -0.04 | -0.20 | 0 | 0 | 0 | -0.35 |
|  |  | RR | -0.13 | -0.05 | -0.21 | 0 | 0 | 0 | -0.38 |
|  |  | FD | -0.15 | -0.04 | -0.18 | 0 | 0 | 0 | -0.37 |
|  | North America | 2015 | -0.08 | -0.04 | -0.23 | 0 | 0 | 0 | -0.36 |
|  |  | S | -0.11 | -0.03 | -0.22 | 0 | 0 | 0 | -0.36 |
|  |  | RR | -0.13 | -0.03 | -0.24 | 0 | 0 | 0 | -0.41 |
|  |  | FD | -0.15 | -0.04 | -0.23 | 0 | 0 | 0 | -0.41 |
|  | North-East Asia | 2015 | -0.08 | -0.02 | -0.58 | 0 | 0 | 0 | -0.69 |
|  |  | S | -0.10 | -0.02 | -0.55 | 0 | 0 | 0 | -0.68 |
|  |  | RR | -0.12 | -0.03 | -0.54 | 0 | 0 | 0 | -0.69 |
|  |  | FD | -0.14 | -0.02 | -0.55 | 0 | 0 | 0 | -0.72 |
|  | Oceania | 2015 | -0.08 | -0.03 | -0.34 | 0 | 0 | 0 | -0.45 |
|  |  | S | -0.10 | -0.03 | -0.33 | 0 | 0 | 0 | -0.46 |
|  |  | RR | -0.13 | -0.03 | -0.33 | 0 | 0 | 0 | -0.49 |
|  |  | FD | -0.15 | -0.03 | -0.32 | 0 | 0 | 0 | -0.49 |
|  | South America | 2015 | -0.08 | -0.04 | -0.35 | 0 | 0 | 0 | -0.47 |
|  |  | S | -0.10 | -0.04 | -0.34 | 0 | 0 | 0 | -0.48 |
|  |  | RR | -0.13 | -0.04 | -0.36 | 0 | 0 | 0 | -0.53 |
|  |  | FD | -0.14 | -0.04 | -0.35 | 0 | 0 | 0 | -0.53 |
|  | South Asia | 2015 | -0.08 | -0.03 | -0.47 | 0 | 0 | 0 | -0.58 |
|  |  | S | -0.10 | -0.03 | -0.46 | 0 | 0 | 0 | -0.59 |
|  |  | RR | -0.13 | -0.03 | -0.50 | 0 | 0 | 0 | -0.66 |
|  |  | FD | -0.14 | -0.03 | -0.48 | 0 | 0 | 0 | -0.65 |
|  | South-East Asia | 2015 | -0.08 | -0.05 | -0.39 | 0 | 0 | 0 | -0.52 |
|  |  | S | -0.10 | -0.04 | -0.39 | 0 | 0 | 0 | -0.53 |
|  |  | RR | -0.13 | -0.05 | -0.40 | 0 | 0 | 0 | -0.58 |
|  |  | FD | -0.14 | -0.04 | -0.40 | 0 | 0 | 0 | -0.59 |
|  | Southern Africa | 2015 | -0.08 | -0.03 | -0.44 | 0 | 0 | 0 | -0.55 |
|  |  | S | -0.10 | -0.03 | -0.42 | 0 | 0 | 0 | -0.55 |
|  |  | RR | -0.13 | -0.02 | -0.56 | 0 | 0 | 0 | -0.70 |
|  |  | FD | -0.14 | -0.03 | -0.43 | 0 | 0 | 0 | -0.60 |
|  | West Africa | 2015 | -0.08 | -0.02 | -0.48 | 0 | 0 | 0 | -0.59 |
|  |  | S | -0.10 | -0.02 | -0.47 | 0 | 0 | 0 | -0.60 |
|  |  | RR | -0.13 | -0.01 | -0.53 | 0 | 0 | 0 | -0.67 |
|  |  | FD | -0.14 | -0.02 | -0.49 | 0 | 0 | 0 | -0.65 |
|  | Western Asia | 2015 | -0.08 | -0.03 | -0.48 | 0 | 0 | 0 | -0.58 |
|  |  | S | -0.10 | -0.02 | -0.47 | 0 | 0 | 0 | -0.60 |
|  |  | RR | -0.13 | -0.03 | -0.47 | 0 | 0 | 0 | -0.62 |
|  |  | FD | -0.14 | -0.03 | -0.46 | 0 | 0 | 0 | -0.63 |
|  | Western Europe | 2015 | -0.08 | -0.03 | -0.38 | 0 | 0 | 0 | -0.48 |
|  |  | S | -0.10 | -0.02 | -0.36 | 0 | 0 | 0 | -0.49 |
|  |  | RR | -0.13 | -0.02 | -0.37 | 0 | 0 | 0 | -0.52 |
|  |  | FD | -0.15 | -0.02 | -0.37 | 0 | 0 | 0 | -0.53 |
|  | World | 2015 | -0.08 | -0.03 | -0.35 | 0 | 0 | 0 | -0.47 |
|  |  | S | -0.10 | -0.03 | -0.34 | 0 | 0 | 0 | -0.48 |
|  |  | RR | -0.13 | -0.03 | -0.36 | 0 | 0 | 0 | -0.52 |
|  |  | FD | -0.15 | -0.03 | -0.35 | 0 | 0 | 0 | -0.52 |
| Vertebrates | Caribbean | 2015 | -0.05 | 0 | -0.28 | -0.07 | -0.08 | -0.03 | -0.51 |
|  |  | S | -0.06 | 0 | -0.25 | -0.08 | -0.16 | -0.03 | -0.57 |
|  |  | RR | -0.07 | 0 | -0.27 | -0.08 | -0.15 | -0.03 | -0.59 |
|  |  | FD | -0.08 | 0 | -0.26 | -0.08 | -0.16 | -0.03 | -0.60 |
|  | Central Africa | 2015 | -0.05 | 0 | -0.15 | -0.02 | -0.02 | -0.21 | -0.45 |
|  |  | S | -0.06 | 0 | -0.14 | -0.04 | -0.05 | -0.19 | -0.49 |
|  |  | RR | -0.07 | 0 | -0.23 | -0.04 | -0.05 | -0.14 | -0.54 |
|  |  | FD | -0.08 | 0 | -0.21 | -0.04 | -0.05 | -0.15 | -0.53 |
|  | Central Asia | 2015 | -0.05 | 0 | -0.47 | -0.03 | -0.03 | 0 | -0.58 |
|  |  | S | -0.06 | 0 | -0.45 | -0.04 | -0.05 | 0 | -0.60 |
|  |  | RR | -0.07 | 0 | -0.45 | -0.04 | -0.05 | 0 | -0.61 |
|  |  | FD | -0.08 | 0 | -0.45 | -0.04 | -0.05 | 0 | -0.62 |
|  | East Africa | 2015 | -0.05 | 0 | -0.33 | -0.03 | -0.03 | -0.09 | -0.53 |
|  |  | S | -0.06 | 0 | -0.31 | -0.06 | -0.06 | -0.08 | -0.57 |
|  |  | RR | -0.07 | 0 | -0.40 | -0.05 | -0.06 | -0.04 | -0.63 |
|  |  | FD | -0.08 | 0 | -0.32 | -0.05 | -0.06 | -0.06 | -0.58 |
|  | Eastern Europe | 2015 | -0.05 | 0 | -0.10 | -0.03 | -0.04 | 0 | -0.22 |
|  |  | S | -0.07 | 0 | -0.10 | -0.04 | -0.04 | 0 | -0.24 |
|  |  | RR | -0.08 | 0 | -0.10 | -0.04 | -0.04 | 0 | -0.25 |
|  |  | FD | -0.09 | 0 | -0.10 | -0.03 | -0.04 | 0 | -0.26 |
|  | Mesoamerica | 2015 | -0.05 | 0 | -0.28 | -0.05 | -0.08 | -0.09 | -0.55 |
|  |  | S | -0.06 | 0 | -0.26 | -0.07 | -0.11 | -0.10 | -0.60 |
|  |  | RR | -0.07 | 0 | -0.27 | -0.05 | -0.11 | -0.08 | -0.59 |
|  |  | FD | -0.08 | 0 | -0.26 | -0.08 | -0.11 | -0.09 | -0.61 |
|  | North Africa | 2015 | -0.06 | 0 | -0.14 | -0.02 | -0.01 | -0.02 | -0.24 |
|  |  | S | -0.06 | 0 | -0.14 | -0.04 | -0.04 | -0.01 | -0.30 |
|  |  | RR | -0.08 | 0 | -0.15 | -0.04 | -0.04 | -0.01 | -0.32 |
|  |  | FD | -0.09 | 0 | -0.13 | -0.04 | -0.04 | -0.01 | -0.31 |
|  | North America | 2015 | -0.05 | 0 | -0.15 | -0.04 | -0.04 | 0 | -0.28 |
|  |  | S | -0.06 | 0 | -0.15 | -0.04 | -0.04 | 0 | -0.30 |
|  |  | RR | -0.08 | 0 | -0.16 | -0.04 | -0.04 | 0 | -0.32 |
|  |  | FD | -0.09 | 0 | -0.15 | -0.04 | -0.04 | 0 | -0.32 |
|  | North-East Asia | 2015 | -0.05 | 0 | -0.41 | -0.03 | -0.05 | 0 | -0.55 |
|  |  | S | -0.06 | 0 | -0.39 | -0.04 | -0.06 | 0 | -0.56 |
|  |  | RR | -0.07 | 0 | -0.39 | -0.04 | -0.06 | 0 | -0.56 |
|  |  | FD | -0.08 | 0 | -0.39 | -0.03 | -0.06 | 0 | -0.57 |
|  | Oceania | 2015 | -0.05 | 0 | -0.26 | -0.03 | -0.02 | -0.02 | -0.38 |
|  |  | S | -0.06 | 0 | -0.24 | -0.06 | -0.05 | -0.02 | -0.43 |
|  |  | RR | -0.07 | 0 | -0.24 | -0.06 | -0.05 | -0.01 | -0.45 |
|  |  | FD | -0.08 | 0 | -0.24 | -0.06 | -0.05 | -0.02 | -0.45 |
|  | South America | 2015 | -0.05 | 0 | -0.23 | -0.04 | -0.04 | -0.13 | -0.48 |
|  |  | S | -0.06 | 0 | -0.22 | -0.05 | -0.07 | -0.13 | -0.52 |
|  |  | RR | -0.07 | 0 | -0.23 | -0.05 | -0.07 | -0.11 | -0.53 |
|  |  | FD | -0.08 | 0 | -0.23 | -0.05 | -0.07 | -0.11 | -0.54 |
|  | South Asia | 2015 | -0.05 | 0 | -0.27 | -0.04 | -0.07 | -0.02 | -0.45 |
|  |  | S | -0.06 | 0 | -0.30 | -0.04 | -0.10 | -0.02 | -0.53 |
|  |  | RR | -0.07 | 0 | -0.34 | -0.04 | -0.10 | -0.01 | -0.56 |
|  |  | FD | -0.08 | 0 | -0.27 | -0.04 | -0.11 | -0.01 | -0.50 |
|  | South-East Asia | 2015 | -0.05 | 0 | -0.22 | -0.04 | -0.04 | -0.17 | -0.52 |
|  |  | S | -0.06 | 0 | -0.23 | -0.04 | -0.10 | -0.16 | -0.59 |
|  |  | RR | -0.07 | 0 | -0.24 | -0.04 | -0.10 | -0.15 | -0.59 |
|  |  | FD | -0.08 | 0 | -0.23 | -0.04 | -0.10 | -0.15 | -0.59 |
|  | Southern Africa | 2015 | -0.05 | 0 | -0.32 | -0.04 | -0.04 | -0.07 | -0.52 |
|  |  | S | -0.06 | 0 | -0.29 | -0.06 | -0.07 | -0.08 | -0.56 |
|  |  | RR | -0.07 | 0 | -0.39 | -0.05 | -0.07 | -0.03 | -0.61 |
|  |  | FD | -0.08 | 0 | -0.31 | -0.06 | -0.07 | -0.07 | -0.58 |
|  | West Africa | 2015 | -0.05 | 0 | -0.32 | -0.02 | -0.02 | -0.06 | -0.47 |
|  |  | S | -0.06 | 0 | -0.29 | -0.04 | -0.06 | -0.06 | -0.51 |
|  |  | RR | -0.07 | 0 | -0.33 | -0.03 | -0.06 | -0.03 | -0.53 |
|  |  | FD | -0.08 | 0 | -0.31 | -0.03 | -0.06 | -0.04 | -0.52 |
|  | Western Asia | 2015 | -0.05 | 0 | -0.34 | -0.02 | -0.03 | 0 | -0.45 |
|  |  | S | -0.06 | 0 | -0.34 | -0.04 | -0.05 | 0 | -0.49 |
|  |  | RR | -0.08 | 0 | -0.34 | -0.04 | -0.05 | 0 | -0.51 |
|  |  | FD | -0.08 | 0 | -0.32 | -0.03 | -0.05 | 0 | -0.48 |
|  | Western Europe | 2015 | -0.05 | 0 | -0.23 | -0.05 | -0.07 | 0 | -0.40 |
|  |  | S | -0.06 | 0 | -0.22 | -0.05 | -0.10 | 0 | -0.44 |
|  |  | RR | -0.07 | 0 | -0.23 | -0.05 | -0.10 | 0 | -0.46 |
|  |  | FD | -0.08 | 0 | -0.23 | -0.05 | -0.10 | 0 | -0.46 |
|  | World | 2015 | -0.05 | 0 | -0.23 | -0.03 | -0.04 | -0.04 | -0.40 |
|  |  | S | -0.06 | 0 | -0.23 | -0.05 | -0.06 | -0.04 | -0.44 |
|  |  | RR | -0.07 | 0 | -0.25 | -0.04 | -0.06 | -0.03 | -0.46 |
|  |  | FD | -0.08 | 0 | -0.23 | -0.04 | -0.06 | -0.04 | -0.45 |
|  |  |  |  |  |  |  |  |  |  |
|  |  |  |  |  |  |  |  |  |  |
|  |  |  |  |  |  |  |  |  |  |
|  |  |  |  |  |  |  |  |  |  |
|  |  |  |  |  |  |  |  |  |  |

**Table S5** Global total area (km^2^) of different land-use types per scenario and changes (%) in area relative to 2015.

| **Land use type** | **2015** | **Sustainability** | | **Regional rivalry** | | **Fossil-fuelled**  **development** | |
| --- | --- | --- | --- | --- | --- | --- | --- |
|  | area | area | change | area | change | area | change |
| urban | 7.4E+05 | 1.2E+06 | 61.3 | 1.0E+06 | 41.2 | 1.3E+06 | 70.1 |
| cropland - minimal use | 2.0E+07 | 1.6E+07 | -18.9 | 1.7E+07 | -13.6 | 2.4E+07 | 17.5 |
| cropland - intense use | 5.8E+06 | 9.4E+06 | 62.2 | 1.1E+07 | 90.6 | 4.9E+06 | -15.5 |
| pasture - minimal use | 2.7E+07 | 2.3E+07 | -13.9 | 2.6E+07 | -0.9 | 2.6E+07 | -2.9 |
| pasture - intense use | 6.1E+06 | 6.8E+06 | 12.4 | 7.4E+06 | 21.9 | 5.7E+06 | -6.7 |
| agriculture - total | 5.9E+07 | 5.5E+07 | -5.4 | 6.2E+07 | 6.2 | 6.0E+07 | 2.5 |
| forestry | 2.9E+06 | 2.9E+06 | -1.0 | 2.9E+06 | -0.5 | 2.9E+06 | -0.4 |
| secondary vegetation | NA | 3.8E+06 | NA | 2.1E+06 | NA | 1.6E+06 | NA |
| natural | 6.7E+07 | 6.6E+07 | -1.6 | 6.1E+07 | -9.0 | 6.3E+07 | -5.3 |

**References**

Abernethy KA, Coad L, Taylor G, Lee ME, Maisels F (2013) Extent and ecological consequences of hunting in Central African rainforests in the twenty-first century. Philosophical Transactions of the Royal Society B-Biological Sciences*,* 368.

Ay JS, Chakir R, Le Gallo J (2017) Aggregated versus individual land-use models: modeling spatial autocorrelation to increase predictive accuracy. Environmental Modeling & Assessment*,* 22, 129-145.

Benítez-López A, Alkemade R, Schipper AM, Ingram DJ, Verweij PA, Eikelboom JAJ, Huijbregts MAJ (2017) The impact of hunting on tropical mammal and bird populations. Science*,* 356, 180-183.

Benítez-López A, Alkemade R, Verweij PA (2010) The impacts of roads and other infrastructure on mammal and bird populations: A meta-analysis. Biological Conservation*,* 143, 1307-1316.

Benítez-López A, Santini L, Schipper AM, Busana M, Huijbregts MAJ (2019) Intact but empty forests? Patterns of hunting-induced mammal defaunation in the tropics. Plos Biology*,* 17, e3000247-e3000247.

Ciesin (2017) Gridded Population of the World, Version 4 (GPWv4): Population Density, Revision 10. Center for International Earth Science Information Network.

Deutsch CA, Tewksbury JJ, Huey RB, Sheldon KS, Ghalambor CK, Haak DC, Martin PR (2008) Impacts of climate warming on terrestrial ectotherms across latitude. Proceedings of the National Academy of Sciences of the United States of America*,* 105, 6668-6672.

Dinerstein E, Olson D, Joshi A *et al.* (2017) An ecoregion-based approach to protecting half the terrestrial realm. Bioscience*,* 67, 534-545.

ESA (2017) Land Cover CCI Product, User Guide Version 2.0, available at: <http://maps.elie.ucl.ac.be/CCI/viewer/download/ESACCI-LC-Ph2-PUGv2_2.0.pdf>.

FAO (2000) Global Forest Resources Assessment 2000. FAO Forestry Department, Rome

Huang X, Xia JH, Xiao R, He T (2019) Urban expansion patterns of 291 Chinese cities, 1990-2015. International Journal of Digital Earth*,* 12, 62-77.

Hudson LN, Newbold T, Contu S *et al.* (2017) The database of the PREDICTS (Projecting Responses of Ecological Diversity In Changing Terrestrial Systems) project. Ecology and Evolution*,* 7, 145-188.

Hudson LN, Newbold T, Contu S *et al.* (2014) The PREDICTS database: a global database of how local terrestrial biodiversity responds to human impacts. Ecology and Evolution*,* 4, 4701-4735.

Hurtt G, Chini L, Sahajpal R, Frolking S, Al. E (in prep.) Harmonization of global land-use change and management for the period 850-2100. Geoscientific Model Development.

Meijer JR, Huijbregts MAJ, Schotten CGJ, Schipper AM (2018) Global patterns of current and future road infrastructure. Environmental Research Letters*,* 13, 064006.

Midolo G, Alkemade R, Schipper AM, Benítez-López A, Perring MP, De Vries W (2019) Impacts of nitrogen addition on plant species richness and abundance: A global meta-analysis. Global Ecology and Biogeography*,* 28, 398-413.

Nunez S, Arets E, Alkemade R, Verwer C, Leemans R (2019) Assessing the impacts of climate change on biodiversity: Is below 2°C enough? Climatic Change*,* 154, 351–365.

Petz K, Alkemade R, Bakkenes M, Schulp CJE, Van Der Velde M, Leemans R (2014) Mapping and modelling trade-offs and synergies between grazing intensity and ecosystem services in rangelands using global-scale datasets and models. Global Environmental Change*,* 29, 223-234.

Popp A, Calvin K, Fujimori S *et al.* (2017) Land-use futures in the shared socio-economic pathways. Global Environmental Change-Human and Policy Dimensions*,* 42, 331-345.

Post E, Forchhammer MC, Bret-Harte MS *et al.* (2009) Ecological dynamics across the Arctic associated with recent climate change. Science*,* 325, 1355-1358.

Redford KH (1992) The empty forest. Bioscience*,* 42, 412-422.

Riahi K, Van Vuuren DP, Kriegler E *et al.* (2017) The Shared Socioeconomic Pathways and their energy, land use, and greenhouse gas emissions implications: An overview. Global Environmental Change-Human and Policy Dimensions*,* 42, 153-168.

Richards P (2018) It's not just where you farm; it's whether your neighbor does too. How agglomeration economies are shaping new agricultural landscapes. Journal of Economic Geography*,* 18, 87-110.

Ripple WJ, Abernethy K, Betts MG *et al.* (2016) Bushmeat hunting and extinction risk to the world's mammals. Royal Society Open Science*,* 3.

Robinson TP, Wint GRW, Conchedda G *et al.* (2014) Mapping the global distribution of livestock. Plos One*,* 9.

Temme AJAM, Verburg PH (2011) Mapping and modelling of changes in agricultural intensity in Europe. Agriculture Ecosystems & Environment*,* 140, 46-56.

**Annex S1 References to data sources used from PREDICTS**

Aben, J.; Dorenbosch, M.; Herzog, S.K.; Smolders, A.J.P.; Van der Velde, G., 2008. Human disturbance affects a deciduous forest bird community in the Andean foothills of central Bolivia. Bird Conservation International 18:363-380.

Albertos, B.; Lara, F.; Garilleti, R.; Mazimpaka, V., 2005. A survey of the epiphytic bryophyte flora in the northwest of the Iberian Peninsula. Cryptogamie 26:263-289.

Bóçon, R., 2010. Riqueza e abundância de aves em três estágios sucessionais da floresta ombrófila densa submontana, Antonina, Paraná. PhD thesis, Universidade Federal do Paraná.

Baral, S.K.; Katzensteiner, K., 2009. Diversity of vascular plant communities along a disturbance gradient in a central mid-hill community forest of Nepal. Banko Janakari 19:43649.

Barlow, J.; Gardner, T.A.; Araujo, I.S.; Avila-Pires, T.C.; Bonaldo, A.B.; Costa, J.E.; Esposito, M.C.; Ferreira, L.V.; Hawes, J.; Hernandez, M.M.; Hoogmoed, M.S.; Leite, R.N.; Lo-Man-Hung, N.F.; Malcolm, J.R.; Martins, M.B.; Mestre, L.A.M.; Miranda-Santos, R.; Nunes-Gutjahr, A.L.; Overal, W.L.; Parry, L.; Peters, S.L.; Ribeiro-Junior, M.A.; Da Silva, M.N.F.; Da Silva Motta, C.; Peres, C.A., 2007. Quantifying the biodiversity value of tropical primary, secondary, and plantation forests. Proceedings of the National Academy of Sciences of the United States of America 104:18555-18560.

Barlow, J.; Mestre, L.A.M.; Gardner, T.A.; Peres, C.A., 2007. The value of primary, secondary and plantation forests for Amazonian birds. Biological Conservation 136:212-231.

Baur, B.; Cremene, C.; Groza, G.; Rakosy, L.; Schileyko, A.A.; Baur, A.; Stoll, P.; Erhardt, A., 2006. Effects of abandonment of subalpine hay meadows on plant and invertebrate diversity in Transylvania, Romania. Biological Conservation 132:261-273.

Berry, N.J.; Phillips, O.L.; Lewis, S.L.; Hill, J.K.; Edwards, D.P.; Tawatao, N.B.; Ahmad, N.; Magintan, D.; Khen, C.V.; Maryati, M.; Ong, R.C.; Hamer, K.C. 2010. The high value of logged tropical forests: lessons from northern Borneo. Biodiversity and Conservation 19:985-997.

Castro-Luna, A.A.; Sosa, V.J.; Castillo-Campos, G. 2007. Bat diversity and abundance associated with the degree of secondary succession in a tropical forest mosaic in south-eastern Mexico. Animal Conservation 10:219-228.

Center for International Forestry Research (CIFOR) 2013. Multidisciplinary Landscape Assessment - Philippines.

Chapman, K.A.; Reich, P.B., 2007. Land use and habitat gradients determine bird community diversity and abundance in suburban, rural and reserve landscapes of Minnesota, USA. Biological Conservation 135:527-541.

CIFOR 2002. Exploring biological diversity, environment and local people's perspectives in forest landscapes: methods for a multidisciplinary landscape assessment. Center for International Forestry Research (CIFOR), Jakarta.

Clarke, F.M.; Rostant, L.V.; Racey, P.A., 2005. Life after logging: post-logging recovery of a neotropical bat community. Journal of Applied Ecology 42:409-420.

Cockle, K.L.; Leonard, M.L.; Bodrati, A.A., 2005. Presence and abundance of birds in an Atlantic forest reserve and adjacent plantation of shade-grown yerba mate, in Paraguay. Biodiversity and Conservation 14:3265-3288.

Craig, M.D.; Hardy, G.E.St.J.; Fontaine, J.B.; Garkakalis, M.J.; Grigg, A.H.; Grant, C.D.; Fleming, P.A.; Hobbs, R.J., 2012. Identifying unidirectional and dynamic habitat filters to faunal recolonisation in restored mine-pits. Journal of Applied Ecology 49:919-928.

Craig, M.D.; Grigg, A.H.; Hobbs, R.J.; Hardy, G.E.St.J., 2014. Does coarse woody debris density and volume influence the terrestrial vertebrate community in restored bauxite mines? Forest Ecology and Management 318:142-150.

Craig, M.D.; Stokes, V.L.; Hardy, G.E.St.J.; Hobbs, R.J., 2015. Edge effects across boundaries between natural and restored jarrah (*Eucalyptus marginata*) forests in south-western Australia. Austral Ecology 40:186-197.

Dallimer, M.; Parnell, M.; Bicknell, J.E.; Melo, M., 2012. The importance of novel and agricultural habitats for the avifauna of an oceanic island. Journal for Nature Conservation 20:191-199.

Dawson, J.; Turner, C.; Pileng, O.; Farmer, A.; McGary, C.; Walsh, C.; Tamblyn, A.; Yosi, C., 2011. Bird communities of the lower Waria Valley, Morobe Province, Papua New Guinea: a comparison between habitat types. Tropical Conservation Science 4:317-348.

De Lima, R.F.; Dallimer, M.; Atkinson, P.W.; Barlow, J., 2013. Biodiversity and land-use change: understanding the complex responses of an endemic-rich bird assemblage. Diversity and Distributions 19:411-422.

De Thoisy, B.; Richard-Hansen, C.; Goguillon, B.; Joubert, P.; Obstancias, J.; Winterton, P.; Brosse, S. 2010. Rapid evaluation of threats to biodiversity: human footprint score and large vertebrate species responses in French Guiana. Biodiversity and Conservation 19:1567-1584.

Doulton, H.; Marsh, C.; Newman, A.; Bird, K.; Bell, M., 2007. Conservation Comores 2005: biodiversity and resource-use assessment and environmental awareness. Final report. University of Oxford/Comorian Centre National de Documentation et Recherche Scientifique.

Laurance, W.F.; Laurance, S.G.W., 1996. Responses of five arboreal marsupials to recent selective logging in tropical Australia. Biotropica 28:310-322.

Farwig, N.; Sajita, N.; Boehning-Gaese, K., 2008. Conservation value of forest plantations for bird communities in western Kenya. Forest Ecology and Management 255:3885-3892.

Fredriksson, G.M.; Danielsen, L.S.; Swenson, J.E., 2007. Impacts of El Nino related drought and forest fires on sun bear fruit resources in lowland dipterocarp forest of East Borneo. Biodiversity and Conservation 16:1823-1838.

Fukuda, D.; Braken Tisen, O.; Momose, K.; Sakai, S., 2009. Bat diversity in the vegetation mosaic around a lowland dipterocarp forest of Borneo. Raffles Bulletin of Zoology 57:213-221.

García, K.P.; Ortiz Zapata, J.C; Aguayo, M.; D'Elia, G., 2013. Assessing rodent community responses in disturbed environments of the Chilean Patagonia. Mammalia 77:195-204.

Garmendia, A.; Arroyo-Rodriguez, V.; Estrada, A.; Naranjo, E.J.; Stoner, K.E., 2013. Landscape and patch attributes impacting medium- and large-sized terrestrial mammals in a fragmented rain forest. Journal of Tropical Ecology 29:331-344.

Gendreau-Berthiaume, B.; Kneeshaw, D.D.; Harvey, B.D., 2012. Effects of partial cutting and partial disturbance by wind and insects on stand composition, structure and growth in boreal mixed woods. Forestry 85:551-565.

Gomes, L.G.L.; Oostra, V.; Nijman, V.; Cleef, A.M.; Kappelle, M., 2008. Tolerance of frugivorous birds to habitat disturbance in a tropical cloud forest. Biological Conservation 141:860-871.

Granjon, L.; Duplantier, J.M., 2011. Guinean biodiversity at the edge: rodents in forest patches of southern Mali. Mammalian Biology 76:583-591.

Grogan, J.; Jennings, S.B.; Landis, R.M.; Schulze, M.; Baima, A.M.V.; Lopes, J.; Norghauer, J.M.; Oliveira, L.R.; Pantoja, F.; Pinto, D.; Silva, J.N.M.; Vidal, E.; Zimmerman, B.L., 2008. What loggers leave behind: impacts on big-leaf mahogany (*Swietenia macrophylla*) commercial populations and potential for post-logging recovery in the Brazilian Amazon. Forest Ecology and Management 255:269-281.

Herrera, J.P.; Wright, P.C.; Lauterbur, E.; Ratovonjanahary, L.; Taylor, L.L., 2011. The effects of habitat disturbance on lemurs at Ranomafana National Park, Madagascar. International Journal of Primatology 32:1091-1108.

Hietz, P. 2005. Conservation of vascular epiphyte diversity in Mexican coffee plantations. Conservation Biology 19:391-399.

Higuera, D.; Wolf, J.H.D., 2010. Vascular epiphytes in dry oak forests show resilience to anthropogenic disturbance, Cordillera Oriental, Colombia. Caldasia 32:161-174.

Hylander, K.; Weibull, H., 2012. Do time-lagged extinctions and colonizations change the interpretation of buffer strip effectiveness? - a study of riparian bryophytes in the first decade after logging. Journal of Applied Ecology 49:1316-1324.

Ishida, H.; Hattori, T.; Takeda, Y., 2005. Comparison of species composition and richness between primary and secondary lucidophyllous forests in two altitudinal zones of Tsushima Island, Japan. Forest Ecology and Management 213:273-287.

Struebig, M.J.; Kingston, T.; Zubaid, A.; Mohd-Adnan, A.; Rossiter, S., 2008. Conservation value of forest fragments to Palaeotropical bats. Biological Conservation 141:2112-2126.

Jung, T.S.; Powell, T., 2011. Spatial distribution of meadow jumping mice (*Zapus hudsonius*) in logged boreal forest of northwestern Canada. Mammalian Biology 76:678-682.

Katovai, E.; Burley, A.L.; Mayfield, M.M., 2012. Understory plant species and functional diversity in the degraded wet tropical forests of Kolombangara Island, Solomon Islands. Biological Conservation 145:214-224.

Kessler, M.; Kessler, P.J.A.; Gradstein, S.R.; Bach, K.; Schmull, M.; Pitopang, R., 2005. Tree diversity in primary forest and different land use systems in Central Sulawesi, Indonesia. Biodiversity and Conservation 14:547-560.

Kessler, M.; Abrahamczyk, S.; Bos, M.; Buchori, D.; Putra, D.D.; Gradstein, S.R.; Hoehn, P.; Kluge, J.; Orend, F.; Pitopang, R.; Saleh, S.; Schulze, C.H.; Sporn, S.G.; Steffan-Dewenter, I.; Tjitrosoedirdjo, S.; Tscharntke, T., 2009. Alpha and beta diversity of plants and animals along a tropical land-use gradient. Ecological Applications 19:2142-2156.

Kittle, A.M.; Watson, A.C.; Chanaka, K.P.H.; Nimalka, S.H.K., 2012. Status and distribution of the leopard in the central hills of Sri Lanka. Cat News 56:28-31.

Lantschner, M.V.; Rusch, V.; Peyrou, C., 2008. Bird assemblages in pine plantations replacing native ecosystems in NW Patagonia. Biodiversity and Conservation 17:969-989.

Lantschner, M.V.; Rusch, V.; Hayes, J.P. 2012. Habitat use by carnivores at different spatial scales in a plantation forest landscape in Patagonia, Argentina. Forest Ecology and Management 269:271-278.

Laurance, W.F.; Laurance, S.G.W., 1996. Responses of five arboreal marsupials to recent selective logging in tropical Australia. Biotropica 28:310-322.

Letcher, S.G.; Chazdon, R.L., 2009. Rapid recovery of biomass, species richness, and species composition in a forest chronosequence in northeastern Costa Rica. Biotropica 41:608-617.

Louhaichi, M.; Salkini, A.K.; Petersen, S.L., 2009. Effect of small ruminant grazing on the plant community characteristics of semiarid Mediterranean ecosystems. International Journal of Agriculture and Biology 11:681-689.

Marin-Spiotta, E.; Ostertag, R.; Silver, W.L., 2007. Long-term patterns in tropical reforestation: plant community composition and aboveground biomass accumulation. Ecological Applications 17:828-839.

Martin, P.S.; Gheler-Costa, C.; Lopes, P.C.; Rosalino, L.M.; Verdade, L.M., 2012. Terrestrial non-volant small mammals in agro-silvicultural landscapes of Southeastern Brazil. Forest Ecology and Management 282:185-195.

Mayfield, M.M.; Ackerly, D.; Daily, G.C., 2006. The diversity and conservation of plant reproductive and dispersal functional traits in human-dominated tropical landscapes. Journal of Ecology 94:522-536.

McNamara, S.; Erskine, P.D.; Lamb, D.; Chantalangsy, L.; Boyle, S., 2012. Primary tree species diversity in secondary fallow forests of Laos. Forest Ecology and Management 281:93-99.

Munyekenye, F.B.; Mwangi, E.M.; Gichuki, N.N., 2008. Bird species richness and abundance in different forest types at Kakamega Forest, western Kenya. Ostrich 79:37-42.

Nakashima, Y.; Inoue, E.; Akomo-Okoue, E.F., 2013. Population density and habitat preferences of forest duikers in Moukalaba-Doudou National Park, Gabon. African Zoology 48:395-399.

Naithani, A.; Bhatt, D., 2012. Bird community structure in natural and urbanized habitats along an altitudinal gradient in Pauri district (Garhwal Himalaya) of Uttarakhand state, India. Biologia 67:800-808.

Nakagawa, M.; Miguchi, H.; Nakashizuka, T. 2006. The effects of various forest uses on small mammal communities in Sarawak, Malaysia. Forest Ecology and Management 231:55-62.

Neuschulz, E.L.; Botzat, A.; Farwig, N., 2011. Effects of forest modification on bird community composition and seed removal in a heterogeneous landscape in South Africa. Oikos 120:1371-1379.

Nicolas, V.; Barriere, P.; Tapiero, A.; Colyn, M., 2009. Shrew species diversity and abundance in Ziama Biosphere Reserve, Guinea: comparison among primary forest, degraded forest and restoration plots. Biodiversity and Conservation 18:2043-2061.

Norfolk, O.; Eichhorn, M.P.; Gilbert, F., 2013. Traditional agricultural gardens conserve wild plants and functional richness in arid South Sinai. Basic and Applied Ecology 14:659-669.

Nöske, N.M.; Hilt, N.; Werner, F.A.; Brehm, G.; Fiedler, K.; Sipman, H.J.M.; Gradstein, S.R., 2008. Disturbance effects on diversity of epiphytes and moths in a montane forest in Ecuador. Basic and Applied Ecology 9:43803.

O'Connor, T.G., 2005. Influence of land use on plant community composition and diversity in Highland Sourveld grassland in the southern Drakensberg, South Africa. Journal of Applied Ecology 42:975-988.

O'Dea, N.; Whittaker, R.J., 2007. How resilient are Andean montane forest bird communities to habitat degradation? Biodiversity and Conservation 16:1131-1159.

Owiunji, I.; Plumptre, A.J., 1998. Bird communities in logged and unlogged compartments in Budongo Forest, Uganda. Forest Ecology and Management 108:115-126.

Paritsis, J.; Aizen, M.A., 2008. Effects of exotic conifer plantations on the biodiversity of understory plants, epigeal beetles and birds in Nothofagus dombeyi forests. Forest Ecology and Management 255:1575-1583.

Parry, L.; Barlow, J.; Peres, C.A., 2009. Hunting for sustainability in tropical secondary forests. Conservation Biology 23:1270-1280.

Peri, P.L.; Lencinas, M.V.; Martínez Pastur, G.; Wardell-Johnson, G.W.; Lasagno, R., 2013. Diversity patterns in the steppe of Argentinean southern Patagonia: environmental drivers and impact of grazing. In: Morales, M.B., Traba Diaz, J (eds), *Steppe Ecosystems*. *Biological Diversity, Management and Restoration*. Nova Science Publishers, UK.

Pincheira-Ulbrich, J.; Rau, J.R.; Smith-Ramirez, C., 2012. Vascular epiphytes and climbing plants diversity in an agroforestal landscape in southern Chile: a comparison among native forest fragments. Boletin De La Sociedad Argentina De Botanica 47:411-426.

Pons, P.; Wendenburg, C., 2005. The impact of fire and forest conversion into savanna on the bird communities of West Madagascan dry forests. Animal Conservation 8:183-193.

Ramos-Robles, M.; Gallina, S.; Mandujano, S., 2013. Habitat and human factors associated with white-tailed deer density in the tropical dry forest of Tehuacan-Cuicatlan Biosphere Reserve, Mexico. Tropical Conservation Science 6:70-86.

Reid, J.L.; Harris, J.B.C.; Zahawi, R.A., 2012. Avian habitat preference in tropical forest restoration in southern Costa Rica. Biotropica 44:350-359.

Sam, K.; Koane, B.; Jeppy, S.; Novotny, V., 2014. Effect of forest fragmentation on bird species richness in Papua New Guinea. Journal of Field Ornithology 85:152-167.

Schumann, K.; Wittig, R.; Thiombiano, A.; Becker, U.; Hahn, K., 2011. Impact of land-use type and harvesting on population structure of a non-timber forest product-providing tree in a semi-arid savanna, West Africa. Biological Conservation 144:2369-2376.

Scott, D.M.; Brown, D.; Mahood, S.; Denton, B.; Silburn, A.; Rakotondraparany, F., 2006. The impacts of forest clearance on lizard, small mammal and bird communities in the arid spiny forest, southern Madagascar. Biological Conservation 127:72-87.

Sedlock, J.L.; Weyandt, S.E.; Cororan, L.; Damerow, M.; Hwa, S.-H.; Pauli, B., 2008. Bat diversity in tropical forest and agro-pastoral habitats within a protected area in the Philippines. Acta Chiropterologica 10:349-358.

Siebert, S.J., 2011. Patterns of plant species richness of temperate and tropical grassland in South Africa. Plant Ecology and Evolution 144:249-254.

Soh, M.C.K.; Sodhi, N.S.; Lim, S.L.H., 2006. High sensitivity of montane bird communities to habitat disturbance in Peninsular Malaysia. Biological Conservation 129:149-166.

Svenning, J.C., 1998. The effect of land-use on the local distribution of palm species in an Andean rain forest fragment in northwestern Ecuador. Biodiversity and Conservation 7:1529-1537.

Wells, K.; Kalko, E.K.V.; Lakim, M.B..; Pfeiffer, M., 2007. Effects of rain forest logging on species richness and assemblage composition of small mammals in Southeast Asia. Journal of Biogeography 34:1087-1099.

Willig, M.R.; Presley, S.J.; Bloch, C.P.; Hice, C.L.; Yanoviak, S.P.; Diaz, M.M.; Chauca, L.A.; Pacheco, V.; Weaver, S.C., 2007. Phyllostomid bats of lowland Amazonia: effects of habitat alteration on abundance. Biotropica 39:737-746.

Yamaura, Y.; Royle, J.A.; Shimada, N.; Asanuma, S.; Sato, T.; Taki, H.; Makino, S., 2012. Biodiversity of man-made open habitats in an underused country: a class of multispecies abundance models for count data. Biodiversity and Conservation 21:1365-1380.

Yoshikura, S.; Yasui, S.; Kamijo, T. 2011. Comparative study of forest-dwelling bats' abundances and species richness between old-growth forests and conifer plantations in Nikko National Park, central Japan. Mammal Study 36:189-198.
